# SIFT-SNN for Traffic-Flow Infrastructure Safety: A Real-Time Context-Aware Anomaly Detection Framework

**DOI:** 10.3390/jimaging12020064

**Published:** 2026-01-31

**Authors:** Munish Rathee, Boris Bačić, Maryam Doborjeh

**Affiliations:** 1 School of Engineering, Computer and Mathematical Sciences, Auckland University of Technology, Auckland 1010, New Zealand; 2 Knowledge Engineering and Discovery Research Innovation, Auckland University of Technology, Auckland 1010, New Zealand

**Keywords:** spiking neural networks, SIFT feature extraction, anomaly detection in infrastructure, context-aware vision systems, spatiotemporal image analysis, edge AI deployment, neuromorphic computing, multi-class defect classification, real-time structural monitoring, intelligent transport systems

## Abstract

Automated anomaly detection in transportation infrastructure is essential for enhancing safety and reducing the operational costs associated with manual inspection protocols. This study presents an improved neuromorphic vision system, which extends the prior SIFT-SNN (scale-invariant feature transform–spiking neural network) proof-of-concept by incorporating temporal feature aggregation for context-aware and sequence-stable detection. Analysis of classical stitching-based pipelines exposed sensitivity to motion and lighting variations, motivating the proposed temporally smoothed neuromorphic design. SIFT keypoints are encoded into latency-based spike trains and classified using a leaky integrate-and-fire (LIF) spiking neural network implemented in PyTorch. Evaluated across three hardware configurations—an NVIDIA RTX 4060 GPU, an Intel i7 CPU, and a simulated Jetson Nano—the system achieved 92.3% accuracy and a macro F1 score of 91.0% under five-fold cross-validation. Inference latencies were measured at 9.5 ms, 26.1 ms, and ~48.3 ms per frame, respectively. Memory footprints were under 290 MB, and power consumption was estimated to be between 5 and 65 W. The classifier distinguishes between safe, partially dislodged, and fully dislodged barrier pins, which are critical failure modes for the Auckland Harbour Bridge’s Movable Concrete Barrier (MCB) system. Temporal smoothing further improves recall for ambiguous cases. By achieving a compact model size (2.9 MB), low-latency inference, and minimal power demands, the proposed framework offers a deployable, interpretable, and energy-efficient alternative to conventional CNN-based inspection tools. Future work will focus on exploring the generalisability and transferability of the work presented, additional input sources, and human–computer interaction paradigms for various deployment infrastructures and advancements.

## 1. Introduction

### 1.1. Background

Traffic-related fatalities remain one of the most urgent public health concerns worldwide, with over 1.25 million deaths and between 20 and 50 million injuries reported annually [1,2,3]. The World Health Organisation forecasts that by 2030, road accidents will become the fifth leading cause of death globally [4]. Beyond the human cost, the global economic burden of road traffic injuries is projected to exceed USD 1.8 trillion from 2015 to 2030, equivalent to an annual tax of 0.12% on the world GDP [5]. Poor road infrastructure, including degraded surfaces, missing safety barriers, and inadequate design, plays a significant role in global road safety failures. According to the UNECE (2023), over 1.19 million people die annually in traffic accidents, 90% of whom are from low- and middle-income countries, with 54% of fatalities involving vulnerable road users such as pedestrians, cyclists, and motorcyclists, groups that are especially exposed due to unsafe or poorly maintained infrastructure [6].

The degrading condition of various traffic infrastructure is amplified by the growing complexity of modern systems, which include over 64 million kilometres of roads and more than one million bridges worldwide [3]. Recent high-profile collapses, such as the Morandi Bridge in Genoa (2018) and the Gujarat pedestrian bridge in India (2022), have highlighted the global vulnerability of aging or mismanaged infrastructure [7,8].

Currently, the predominant model for structural health monitoring is periodic manual inspection, with cycles ranging from six months to five years, depending on regional regulations [9]. While historically effective, this approach is increasingly unfit for modern demands due to high labour and safety-associated costs, subjective variability, exposure risks, and its inability to detect sudden or progressive failures between inspection windows.

In response to the limitations of manual inspections, there has been growing interest in Automated Road Defect and Anomaly Detection (ARDAD) systems that integrate computer vision (CV), machine learning (ML), and deep learning (DL) to automate large-scale inspection workflows [10,11]. A common objective guiding automation in ARDAD is the intention to reduce human dependency, enable real-time alerts, and enhance coverage. However, the current ARDAD landscape remains disproportionately focused on surface-level features, such as potholes, cracks, and asphalt delamination, while neglecting embedded or mechanical anomalies that may not manifest through traditional image cues yet often precede catastrophic structural failures [11].

### 1.2. Structural Anomalies in Context

Contextual or structural anomalies, such as displaced manhole covers, missing safety bolts, or modular component failure, often lack prominent surface indicators yet pose a disproportionate threat to structural integrity and user safety. For example, investigations commonly focus on out-of-place components within highly regular, load-bearing structures.

One critical example is the Movable Concrete Barrier (MCB) system installed on the Auckland Harbour Bridge (AHB), a vital multi-span transport corridor that carries more than 170,000 vehicles per day [12]. The bridge employs a barrier transfer machine (BTM) to reposition concrete segments across lanes based on traffic flow demand [13]. Mechanical metal pins secure each segment; consequently, the failure or partial dislodgement of any of the barrier components may result in severe misalignment or the progressive collapse of the barrier sections.

**Figure 1:**
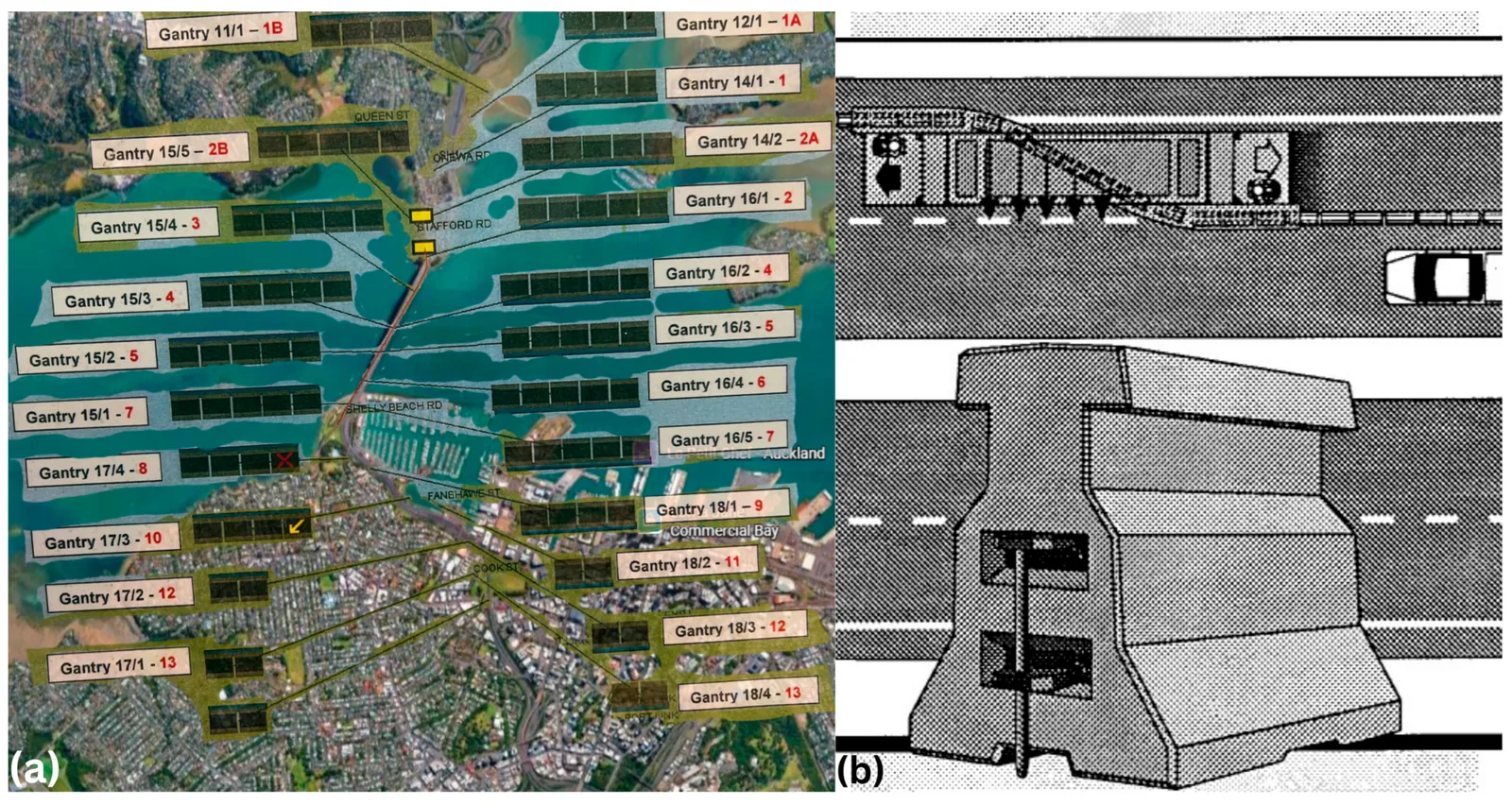
(**a**) Gantry distribution and control zones for the movable concrete barrier (MCB) system along the Auckland Harbour Bridge corridor, showing active BTM-managed segments and gantry identifiers (Courtesy of Google Maps v2025). (**b**) Schematic representation of the barrier transfer machine (BTM) repositioning MCB units, with an inset showing the metal pin linkage between adjacent barrier blocks essential for structural continuity (Source: Cottrell [14]).

Currently, inspections of barrier pins are conducted manually, and they are only performed during off-peak hours. No embedded or automated system exists for continuous monitoring, creating potential blind spots in infrastructure safety. Figure 1 illustrates the operational layout of the BTM system and its dependence on the structural integrity of the MCB units.

Given the mechanical and spatial regularity of such systems, context-aware detection approaches—those capable of understanding expected structural configurations over time—are essential for identifying subtle, high-risk deviations.

**Figure 2:**
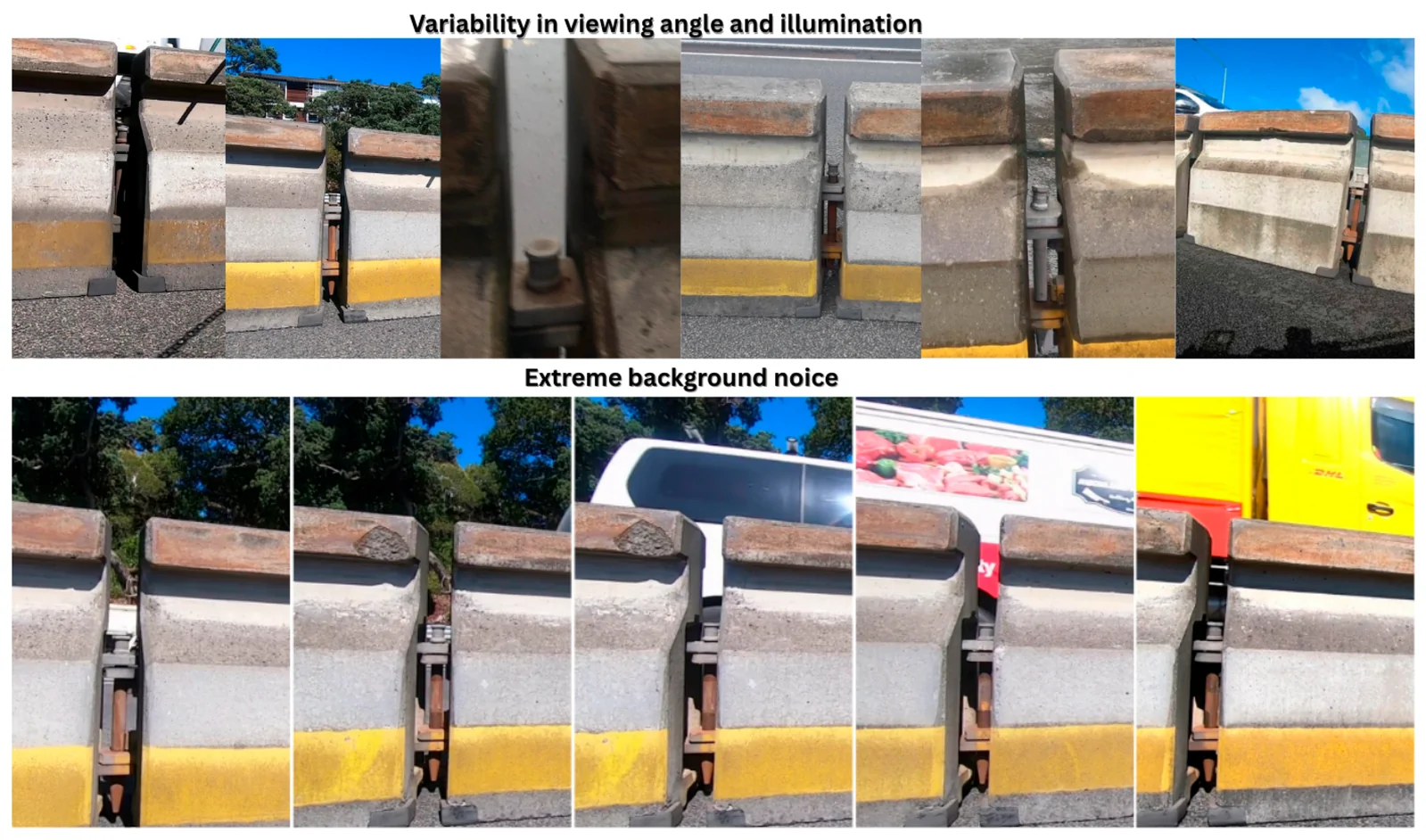
Representative inspection frames from the Auckland Harbour Bridge Movable Concrete Barrier system, illustrating variability in viewing angle, illumination, and background conditions caused by live traffic during operation.

In practice, inspection imagery is captured under highly variable conditions, including changes in viewing angle, illumination, and background dynamics caused by live traffic. As illustrated in Figure 2, these factors create a visually unstable environment in which subtle mechanical anomalies must be detected reliably, motivating the need for robust feature representations and temporal reasoning.

### 1.3. Motivational Gaps in Existing Systems

Although recent advances in image processing, computer vision (CV), and general AI-enabled inspections have yielded promising results in surface-level defect detection, significant limitations persist when applied to complex transport infrastructure such as the Auckland Harbour Bridge. The following shortcomings remain particularly unresolved:

- Lack of subsurface and structural sensitivity: The majority of Automated Road Defect and Anomaly Detection (ARDAD) systems are designed to detect visible surface irregularities, such as cracks and potholes. These models are not equipped to identify embedded or mechanical anomalies, such as displaced pins or loose joints, that may not be visually salient but pose critical safety risks.
- Dependence on extensive training data: Deep learning-based solutions typically require large, annotated datasets that capture diverse operational scenarios. Domain shift and environmental variability (e.g., lighting, motion blur, and weather) often reduce model generalisation in real-world deployments.
- Limited temporal reasoning: Most systems operate on a frame-by-frame basis, ignoring temporal continuity. As a result, they are incapable of detecting progressive or transient anomalies that unfold over time, a key requirement for dynamic infrastructure such as movable barriers.
- High computational complexity: State-of-the-art CNN architectures, while accurate, are computationally intensive and often require significant GPU resources. This makes them unsuitable for embedded or roadside units that must operate in real time under constrained power and latency budgets. Spatiotemporal models developed for video anomaly detection further highlight this limitation, as they typically operate on dense frame sequences and rely on heavy CNN or transformer backbones, which hinder real-time deployment on resource-constrained embedded hardware [15,16].
- Low granularity in classification: Many ARDAD frameworks offer binary classification outputs (defect/no defect), which are insufficient for infrastructure systems that require graduated assessments of failure states (e.g., safe, partial displacement, total failure).

While some attempts have been made to preserve spatial context, such as frame aggregation under motion to merge adjacent frames, commonly used methods are unreliable under inspection scenarios involving high-speed motion, parallax distortions, or structural occlusions (Table 1). Recent infrastructure-inspection efforts have attempted dual-sensor image stitching and fusion to cover large spans of structural assets, combining visible and infrared modalities to improve defect visibility [17,18]. However, these approaches rely on consistent overlap and stable imaging conditions, making them vulnerable to motion blur, parallax, and lighting variability—conditions common in movable barrier inspections. As such, they fail to provide stable or coherent representations in contexts like the barrier transfer machine (BTM) traversal along the Auckland Harbour Bridge.

To summarise, existing systems lack the spatiotemporal awareness, structural interpretability, and computational efficiency required for real-time, context-aware safety monitoring of mechanical infrastructure.

### 1.4. Preliminary Work

This study extends our earlier work presented in [19], in which a hybrid SIFT–SNN model combining the scale-invariant feature transform (SIFT) with spiking neural networks (SNN) approaches was introduced as proof-of-concept for the binary anomaly detection of mechanical pin dislodgement on the Auckland Harbour Bridge (AHB). While the initial proof-of-concept demonstrated the feasibility of combining classical descriptors with neuromorphic classification, it was limited in three key aspects:

(i) The absence of temporal modelling.
(ii) A binary decision space that could not capture intermediate or ambiguous states.
(iii) Lack of evaluation under hardware-constrained or real-time operating conditions.

To address the initial scope limitations and progress towards a deployable prototype, we now present a substantially extended system with the following contributions:

- A temporal enhancement layer for modelling inter-frame dependencies.
- A multi-class classification scheme capable of distinguishing between fully safe, partially dislodged, fully dislodged, and ambiguous mechanical pin states.
- A hardware-aware performance evaluation, demonstrating low-latency classification using CPU-only and edge-constrained configurations.

Experience with panorama stitching for spatial aggregation revealed that conventional image-compositing approaches are unstable under motion, parallax, and illumination variations. This limitation motivated the transition from purely spatial aggregation to spatiotemporal feature modelling. The extended system presented here incorporates temporal reasoning, hardware-aware design, and multi-level diagnostic outputs into a unified framework for neuromorphic anomaly detection in smart infrastructure monitoring.

### 1.5. Objectives and Research Questions

This study addresses the limitations of the state of the art (Section 1.3) by advancing a previously validated SIFT-SNN anomaly detection pipeline into a context-aware, deployable system suitable for real-time infrastructure monitoring. The key objectives of the study are as follows:

- To incorporate temporal feature aggregation into the SIFT-SNN framework, enabling spatiotemporal context awareness across sequential inspection frames.
- To extend the anomaly detection task from binary classification to a multi-class recognition scheme, enabling nuanced differentiation between partial, full, and ambiguous defect states.
- To empirically benchmark the system’s latency, diagnostic accuracy, and suitability for edge deployment under constrained hardware deployment conditions.
- To reflect on unsuccessful spatial context integration approaches, including panorama stitching, and clarify their limitations in dynamic transport inspection environments.

The study objectives reflect a multidimensional expansion of the original framework, encompassing the spatial, temporal, and operational dimensions of anomaly detection. By addressing both the methodological enhancements and their real-world constraints, the study aligns technical innovation with practical applicability in safety-critical infrastructure scenarios.

As a contribution to general computer vision and AI, the primary research questions (RQ) guiding the experimental work supporting the study are as follows:

- RQ1: Does the integration of temporal features across frame sequences improve anomaly detection performance over static SIFT-SNN pipelines?
- RQ2: Can the enhanced SIFT-SNN framework operate with real-time latency and maintain diagnostic precision under edge deployment constraints?
- RQ3: To what extent does multi-class classification improve safety diagnostics in infrastructure systems with graded failure modes?

For completeness, we note that early exploration of panorama stitching highlighted challenges in motion-heavy inspection scenarios. These observations motivated the emphasis on temporal modelling within the main research questions, rather than forming a standalone objective.

## 2. Related Work

Advancements in automated infrastructure monitoring have gained significant traction over the past decade and a half (2010–2025), particularly in the domains of road defect detection and structural health diagnostics. A substantial body of research has explored computer vision and deep learning approaches for identifying surface-level anomalies such as cracks, potholes, and material degradation. However, limited attention has been paid to the detection of structural or modular anomalies, particularly those associated with movable or load-bearing components such as mechanical joints, articulating hinges, or safety pins. To identify the critical limitations that motivate the present study, this section reviews existing literature across four key themes: (1) automated road and bridge inspections, (2) structural health monitoring of modular systems, (3) spiking neural networks (SNNs), and (4) temporal modelling and contextual awareness.

### 2.1. Automated Road and Bridge Inspections

Automated Road Defect and Anomaly Detection (ARDAD) systems have evolved significantly over the past two decades, progressing from rule-based image processing and classical computer vision (CV) algorithms to machine learning (ML) and deep learning (DL) frameworks [11]. Traditional approaches, such as edge detection, Hough transforms, and texture analysis, were once dominant in detecting linear cracks and surface disruptions on pavements and bridges. However, traditional approaches for infrastructure anomaly detection (for safety monitoring) often lacked robustness under varying lighting, occlusion, and weather conditions [20].

The rise of convolutional neural networks (CNNs) ushered in a new generation of ARDAD systems capable of learning high-level visual features directly from annotated data. Studies such as Zhang et al. [21] demonstrated the superior performance of deep CNNs in identifying fine-grained crack patterns with high accuracy across diverse road textures. Similarly, Maeda et al. [22] introduced one of the earliest large-scale annotated datasets for road damage detection in Japan, enabling the benchmarking of supervised DL models for crack, pothole, and road marking degradation classification.

More recent research has incorporated drone-based and LiDAR-equipped inspection platforms to improve scene coverage and 3D context capture, particularly for bridges and elevated structures [23,24]. While the most recent advances have significantly improved detection rates and operational scalability, the dominant focus remains on static, surface-visible defects, such as longitudinal cracking, surface rutting, potholes, and material delamination, while deeper structural components are often overlooked [25].

Despite the growing volume of research and the availability of publicly accessible datasets (e.g., Crack500 [21], RDD2020/2022 [22], BDD100K [26], and AigleRN [27]), there remains a notable blind spot in the detection of embedded or structural anomalies. Examples include mechanical joints, fasteners, and movable modules, which often lack visual distinctiveness and require contextual or temporal analysis for reliable detection. Furthermore, the diagnostic emphasis in most existing literature has been on the classification of known surface defects, with limited capability to detect out-of-distribution anomalies or to interpret evolving structural conditions that may not exhibit immediate surface cues.

The identified need for context-aware anomaly detection systems that integrate spatial geometry, temporal dynamics, and neuromorphic efficiency capabilities is the key element to justify the experimental work that led to the creation of the SIFT-SNN framework.

### 2.2. Structural Health Monitoring of Modular Systems

While traditional structural health monitoring (SHM) has primarily targeted large-scale anomalies, such as bridge deck corrosion [28], cable tension loss [29], or girder deformation [30], recent research has begun to address the nuanced challenges posed by modular and movable systems in transport infrastructure [31]. Examples include mechanically interlocked or articulated components, such as expansion joints [32], movable barrier systems [33], hinges, pin assemblies, and rail switches [34], whose failure modes are often subtle and lack distinct visual signatures when observed in isolated frames.

Many conventional SHM methods rely on vibration analysis, strain gauge readings, or acoustic emissions, which, although precise, require embedded sensors and are not always feasible for high-frequency, wide-area inspection [35]. As an alternative, vision-based SHM for modular systems has emerged, leveraging image and video feeds to detect alignment shifts, displacement patterns, or anomalous interactions between moving parts [36]. However, modular systems introduce two persistent challenges that complicate visual diagnostics:

1. Lack of large, annotated datasets for such component-level defects.
2. Need for context-aware modelling due to geometric regularity and positional dependency.

In the context of movable concrete barrier (MCB) systems such as those on the Auckland Harbour Bridge (AHB), the failure of steel connecting pins may not result in overt visual damage but rather in relatively slight misalignments or motion irregularities, which are difficult to detect with static image classifiers [37]. As such, temporal inspection methods, combining structural priors with dynamic context, are increasingly necessary.

Emerging works have proposed using techniques such as the following:

- Three-dimensional reconstruction or photogrammetry to assess displacement over time [38].
- Graph-based modelling of modular units and their interconnections [39].
- Multi-view visual inspection to resolve ambiguous or occluded parts [40].

To date, however, few of the structural health monitoring approaches have been put into operation for edge-deployable or real-time systems suitable for live traffic scenarios. While mapping data and output classes directly to the traffic scenario seems an intuitive approach, most of the existing systems are not designed to handle multi-class or additional states of degradation (e.g., safe, partially failed, fully failed), which is essential for risk-sensitive components. One of the insights guiding our experimental research is based on a rationale for automated barrier pin position monitoring, where false positive vs. false negative errors are not to be treated equally.

To address the gaps identified, this study seeks to build on the preliminary work and insights from anomaly detection in ARDAD systems and extend them with a hybrid neuromorphic vision architecture designed to deliver interpretable, multi-class classification and spatiotemporal reasoning for diagnosing failures in safety-critical structural components.

### 2.3. Spiking Neural Networks (SNNs) in Vision-Based Anomaly Detection

Spiking neural networks (SNNs) constitute the third generation of neural models, incorporating biologically inspired, asynchronous, and event-driven mechanisms for processing neural information. In contrast to conventional deep learning models, such as CNNs or Transformers, which rely on dense, synchronous matrix operations, SNNs transmit information via discrete spikes, emulating the neural dynamics observed in biological systems [41].

Considering increasing expectations for low energy consumption and low computational demands of SNN, this represents a particularly attractive research avenue in CV for real-time, low-power applications in domains such as robotics, neuromorphic vision, and edge-based anomaly detection [42]. The leaky integrate-and-fire (LIF) neuron model remains one of the most widely adopted in SNN implementations due to its simplicity and computational efficiency [43].

**Key Advantages in Infrastructure Monitoring Contexts**

SNNs exhibit intrinsic properties that make them particularly well-suited to safety-critical infrastructure diagnostics:

- Event-driven efficiency: Only responds to salient input events, drastically reducing computation and energy demands [42].
- Temporal sensitivity: Intrinsically models time-series or spatiotemporal data, aligning with the needs of progressive or transient anomaly detection [44].
- Hardware deployability: Compatible with neuromorphic chips (e.g., Loihi, SpiNNaker), making real-time deployment feasible at the edge [45].

The benefits of SNNs have therefore motivated researchers to explore their use in a range of application domains beyond neuroscience and robotics.

**Recent Applications in Safety Inspection**

Although still in its early stages of development in civil infrastructure monitoring, SNN-based models are being gradually introduced into safety-critical tasks. Several recent studies illustrate their promise:

- Jiang et al. used SNNs for surface-level defect classification in noisy visual environments [46].
- Vemuru proposed a biologically inspired spiking neural network (SNN) edge detector employing Gabor-type filters for feature extraction, demonstrating that such hybrid architectures enhance edge saliency in low-exposure conditions and support improved classification in conjunction with CNNs [47].
- In autonomous driving and dynamic scene analysis, SNNs have been shown to outperform CNNs in terms of latency and energy efficiency under constrained compute budgets [48].

However, most existing studies limit SNNs to static input modalities or binary classification settings. Few, if any, have applied SNNs to the following:

- Context-aware multi-class diagnostics in modular infrastructure.
- Progressive fault detection using frame sequences.
- Integration with traditional descriptors (e.g., SIFT) for hybrid pipelines.

This paper builds upon our early proof-of-concept that introduced a hybrid SIFT-SNN system for binary anomaly detection on movable barrier pins [19]. In this paper, the initial proof-of-concept is extended through the integration of temporal modelling, spatiotemporal context fusion, and multi-class diagnostic interpretation, hence marking one of the first attempts to deploy such a hybrid SIFT-SNN architecture in structural safety diagnostics under real-world dynamic conditions.

### 2.4. Temporal Modelling and Contextual Awareness

Contemporary anomaly detection systems, particularly in domains such as surveillance, human activity recognition, and medical diagnostics, have increasingly leveraged temporal cues to improve robustness, context awareness, and failure anticipation [49].

In computer vision, temporal modelling is typically achieved through 3D convolutional networks (C3D), recurrent neural networks (RNNs), temporal attention modules, or optical flow-based methods. For instance, Tran et al. demonstrated that 3D CNNs significantly outperformed 2D models for human action recognition by capturing spatiotemporal features across frames [50]. Likewise, Feichtenhofer et al. introduced the SlowFast network, which processes both slow and fast temporal dynamics, enabling fine-grained motion interpretation [51].

However, such approaches are rarely translated into infrastructure inspection tasks. Most ARDAD or SHM models continue to operate at a per-frame level, overlooking valuable inter-frame dependencies [31]. This omission is particularly problematic in modular systems like movable concrete barriers (MCBs), where subtle misalignments or component displacements only become evident over time [33].

Efforts to bridge spatial discontinuities across video sequences have explored panorama stitching using keypoint-based methods such as SIFT, SURF, or ORB [52,53]. While effective under controlled conditions, keypoint-based methods often fail in real-world inspection scenarios due to the following factors:

- Rapid egomotion of the camera system.
- Structural parallax between bridge segments.
- Occlusion and lighting variations.
- Perspective distortions and lens warp.

The limitations of keypoint-based methods significantly undermine the reliability of stitched spatial representations, especially in edge-deployable systems where compute and latency budgets are constrained, computational resources are limited, and real-time performance is crucial [54]. In practice, preliminary tests and visual inspection revealed that these techniques exhibit consistent failure modes when applied to dynamic bridge inspection footage.

Thus, there is a compelling need to develop hybrid approaches that:

- Aggregate temporal cues without relying solely on optical alignment
- Provide interpretable outputs across evolving frame sequences
- Support real-time deployment under hardware constraints.

The above challenges motivate the integration of lightweight temporal modules within a neuromorphic SNN pipeline, enabling both temporal sensitivity and energy efficiency, which, in this paper, we consider hallmarks of robust infrastructure diagnostics. By embedding temporal aggregation directly into a hybrid SIFT-SNN architecture, the proposed system aims to capture evolving structural anomalies while avoiding the computational overhead associated with optical alignment or dense spatiotemporal networks.

### 2.5. Gap Summary and Positioning

The preceding review reveals critical limitations in current automated infrastructure monitoring systems. While surface-level defect detection has advanced substantially, driven by large annotated datasets and deep convolutional architectures, existing models still fall short in several contexts, such as the following:

- Neglect of structural and modular components: Most ARDAD and SHM systems are optimised for surface anomalies and lack sensitivity to structural faults in mechanically interlocked or movable elements, such as barrier pins and joints.
- Lack of temporal reasoning: Per-frame classification approaches dominate the literature, often failing to capture progressive or transient anomalies that unfold over time.
- Limited edge-computing deployability: Deep CNNs and optical flow models typically require significant compute resources, making them impractical for real-time, low-power roadside units.
- Underutilisation of neuromorphic architectures: Despite the clear advantages of SNNs in energy efficiency and temporal encoding, their application to modular infrastructure inspection remains underexplored, particularly in multi-class or context-aware diagnostic tasks.

To address the current technology gaps, this study proposes a novel hybrid SIFT-SNN pipeline, extended with:

- Temporal modelling across frame sequences for progressive anomaly detection;
- Panoramic stitching evaluation under real-world egomotion and structural parallax conditions;
- Multi-class classification of mechanical states (e.g., safe, partial, failed, and ambiguous);
- Latency and power benchmarking on edge-deployable neuromorphic hardware platforms.

Table 2 summarises the comparative positioning of the proposed method against the state of the art across five key dimensions.

The comparative summary of the state-of-the-art positioning underscores the novelty of the proposed framework as one of the first real-time, interpretable, and multi-class SNN-based systems applied to structural anomaly detection in modular infrastructure. Hence, the combined capabilities of the proposed SIFT-SNN framework (Table 2) provide strong supporting arguments for live deployment scenarios on the Auckland Harbour Bridge.

## 3. Materials and Methods

This section presents the experimental design used to develop and evaluate the proposed context-aware SIFT-SNN anomaly detection system for structural safety diagnostics in modular transport infrastructure. The study leverages both a synthetic dataset, designed to simulate graded pin displacement anomalies under controlled conditions, and real-world video footage of the Auckland Harbour Bridge’s movable concrete barrier (MCB Location: Auckland, NZ) system, used for qualitative validation and scenario benchmarking.

We first describe the construction of the synthetic dataset, including image generation protocols, defect labelling schema, and class balance strategies. Next, we outline the preprocessing pipeline, which includes greyscale normalisation, SIFT-based feature extraction, and spike encoding for neuromorphic processing. The hybrid pipeline architecture, comprising a handcrafted feature extractor and a leaky integrate-and-fire (LIF) spiking neural network, is then detailed, followed by the temporal modelling extension used to integrate context across sequential frames.

Finally, we describe the anomaly classification and performance evaluation protocol, including multi-class confusion metrics, latency measurements under CPU-only constraints, and qualitative assessments of real-world footage. As part of a broader methodological reflection, we also report on a failed panorama stitching attempt, included here to illustrate the limitations of classical spatial alignment techniques under high-motion, occlusion-prone conditions.

### 3.1. Data Collection and Ground Truth Annotation

To support the development of a robust and context-aware anomaly detection framework for the Auckland Harbour Bridge’s Movable Concrete Barrier (MCB) system, a composite dataset was constructed using real-world inspection footage and synthetically generated defect cases.

#### 3.1.1. Real-World Data Acquisition

Three supervised field deployments were conducted using a custom rig mounted on the barrier transfer machine (BTM). Multi-angle video footage of the barrier’s metal pin interfaces was captured using consumer-grade cameras (GoPro 5/8/9, GoPro, Inc., San Mateo, CA, USA; iPhone 13 Pro, Apple Inc., Cupertino, CA, USA; Samsung A7, Samsung Electronics, Suwon, Republic of Korea; iPad 6, Apple Inc., Cupertino, CA, USA). Devices were affixed to the BTM’s front and rear arms using vibration-dampening measures and powered externally to support prolonged high-frame-rate recording (up to 240 fps). Camera configurations and deployment conditions are summarised in Table 3.

Environmental variability included the following:

- Lighting conditions: early morning, midday sun, overcast, night-time.
- Motion: slow traversal, full-speed operation, and stop–start transitions.
- Visual noise: structural shadows, cable occlusions, and surface glare.

The data collection recordings (Table 3) were primarily used for qualitative system validation, temporal stability checks, and failure analysis under deployment-like conditions.

**Ground Truth Annotation**

Individual video frames were manually annotated using *LabelMe* (v5.3) and internal Python tools. Each instance was labelled into one of four mutually exclusive alignment states:

- “Safe” … Pins fully seated and flush with block surfaces.
- “Partial Misalignment” … Detectable tilt or partial protrusion.
- “Out” … Significant dislocation or complete disengagement.
- “Ambiguous” … Frames obscured by blur, occlusion, or poor lighting.

Annotations included the following:

- Bounding box coordinates of the region of interest (ROI).
- Class label.
- Environmental metadata (lighting, obstruction, and session ID).

Annotation reliability was confirmed through a dual review, with an inter-rater agreement of greater than 95% on a stratified subset.

#### 3.1.2. Synthetic Frame Augmentation

Because Full Misalignment cases are naturally scarce under normal operation, a procedurally controlled augmentation pipeline was employed to enrich this underrepresented failure class. Uncontrolled recording of pinout conditions during live operation is prohibited under NZTA safety regulations. To mitigate this constraint, a limited set of real failure instances was recorded under supervised conditions, in which authorised Auckland Harbour Bridge staff manually removed selected pins, and the resulting pin-out states were captured. These real pin-out instances serve as the basis for all synthetic augmentation used in this study.

**Figure 3:**
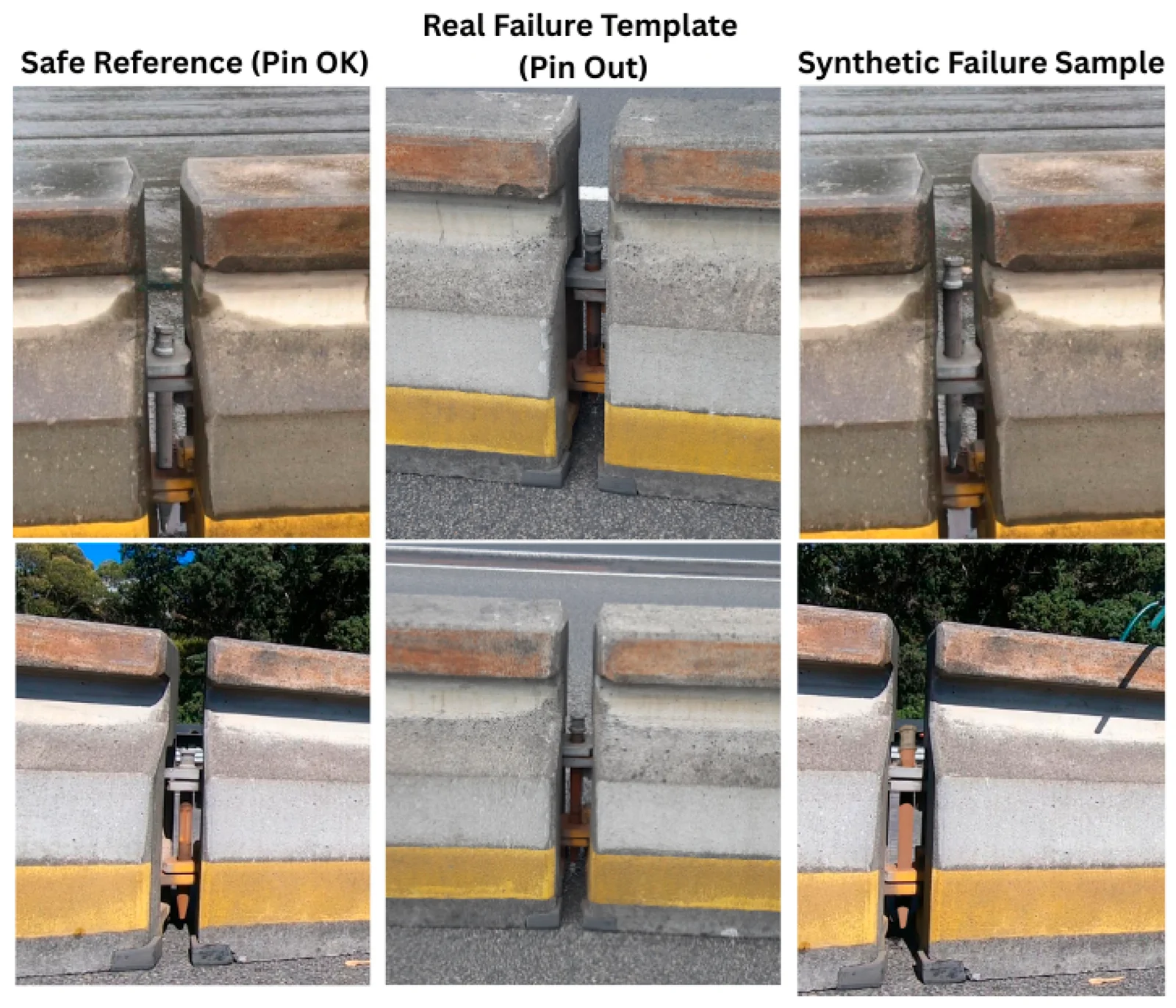
Template-based synthetic augmentation using real pin-out conditions. Shown are safe reference frames (**left**), real failure templates (**centre**), and corresponding synthetic samples (**right**), preserving failure geometry to address class imbalance in rare safety-critical cases.

Building on our previous work [11], the augmentation pipeline applies localised affine transformations, motion blur kernels, and pixel intensity perturbations to generate additional Full Misalignment samples while preserving the true failure location, mechanical alignment, and visual context demonstrated by real pin dislodgement events. As a result, synthetic frames are physically grounded and resemble real failure configurations rather than hypothetical or unconstrained patterns. Representative examples of a safe reference state, a real pin-out template, and the corresponding synthetic failure sample are illustrated in Figure 3.

To minimise dataset imbalance and reduce the risk of domain shift, augmentation was applied under the following constraints:

- Augmentation was limited strictly to the Full Misalignment class.
- The synthetic-to-real ratio was capped at 30% of the total dataset.
- No alterations were applied to other well-represented classes.

This approach preserved statistical integrity while improving the model’s sensitivity to rare but safety-critical anomalies.

### 3.2. Preprocessing Pipeline

The raw field footage and synthetic frame sequences underwent a structured preprocessing pipeline designed to extract stable, discriminative features for neuromorphic inference. The pipeline included temporal segmentation, region-of-interest enhancement, and SIFT-based keypoint extraction, enabling both spatial sparsity and robustness under deployment conditions.

#### 3.2.1. Temporal Windowing and Segmentation

To incorporate short-term motion context and suppress transient noise, video sequences were segmented into overlapping windows of three to five frames. This technique promotes temporal smoothing, aids in ambiguous state disambiguation, and aligns with event-driven temporal modelling approaches common in neuromorphic vision systems [55].

To incorporate short-term motion context and suppress transient classification noise, each video sequence was segmented into overlapping windows of three to five frames. Within these windows (containing frame sequence), spatial features were extracted per frame and then aggregated through temporal smoothing mechanisms embedded in the downstream spike-based architecture. The overlapping window method helps to resolve ambiguous or noisy frames, caused by motion blur, occlusion, or lighting disruptions, by anchoring them to nearby, higher-confidence observations.

**Figure 4:**
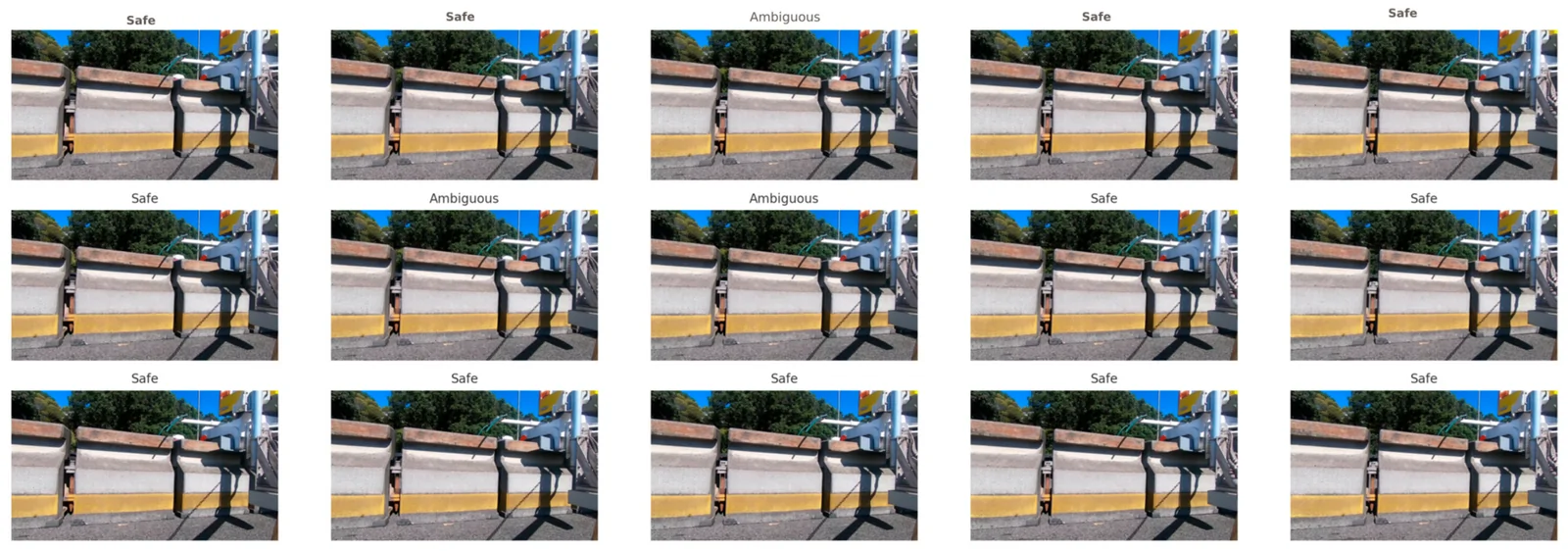
Temporal windowing improves label consistency across frame sequences. While frame-level predictions misclassify blurred or occluded frames as “Ambiguous,” the surrounding temporal context enables correction to “Safe,” demonstrating improved stability for anomaly detection.

Figure 4 depicts the effect of “Ambiguous’ frames” appearance. For example, in a scenario involving partial occlusion, isolated frame classification fails to confidently assign labels to frames that are blurred or darkened. However, when processed within a 3-frame window, temporal dependencies allow contextual correction, resulting in more stable and accurate anomaly classification. This design mirrors the principles of event-driven neuromorphic processing, where temporal sparsity and continuity are leveraged for robust perception.

#### 3.2.2. Region-of-Interest (ROI) Extraction and Enhancement

Each frame was cropped to a fixed ROI bounding box centred on the pin-hinge junction. Following this, the following image enhancement procedures were applied:

- Greyscale conversion to reduce computational complexity and ensure SIFT feature stability under varying lighting [56].
- Contrast Limited Adaptive Histogram Equalisation (CLAHE) to enhance contrast in shaded or overexposed regions locally [57].
- Gaussian blurring (*σ* = 1.2–1.5) to reduce high-frequency noise and suppress spurious keypoint activation during SIFT extraction [56].

**Figure 5:**
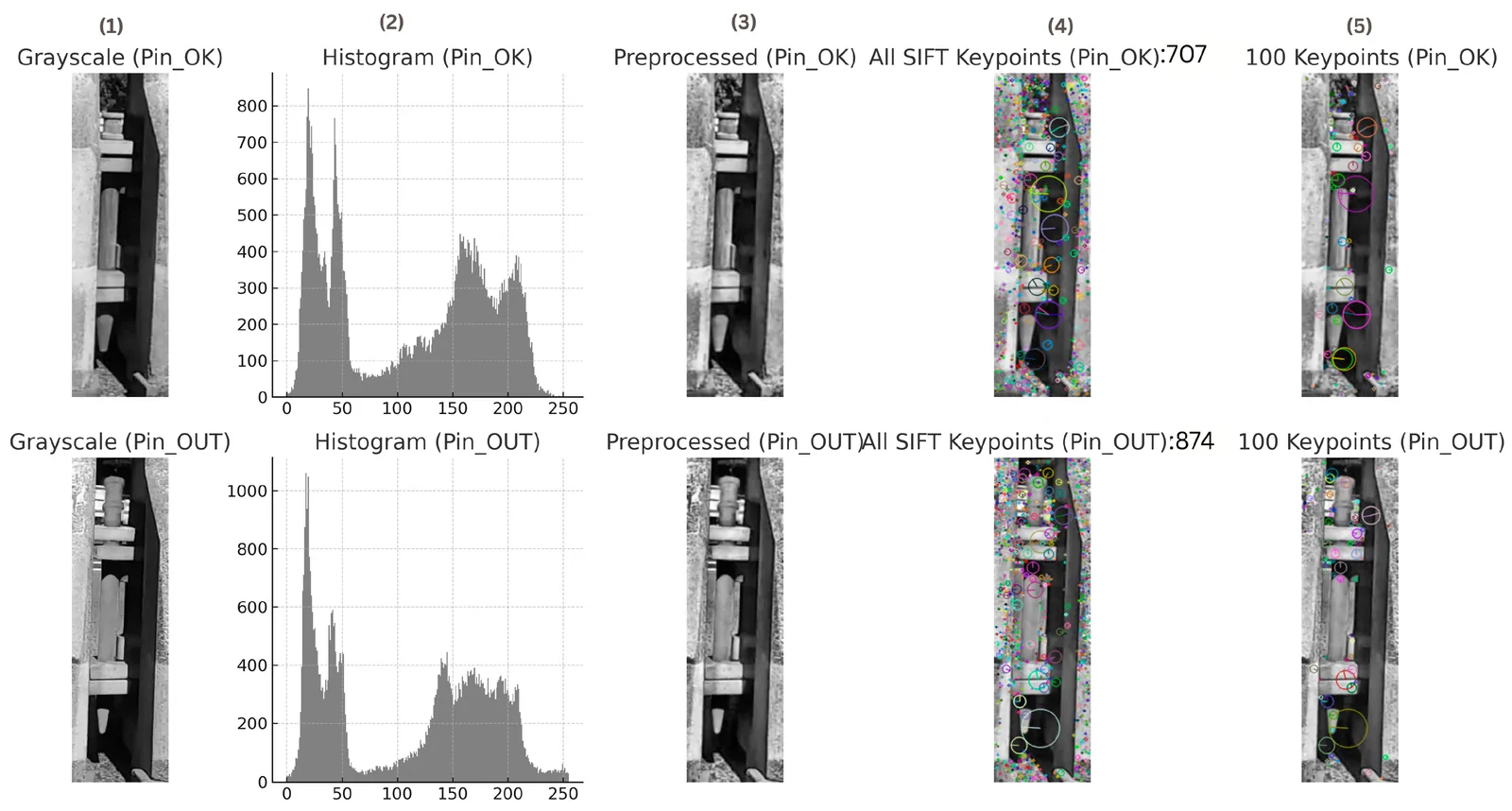
SIFT preprocessing comparison for Pin_OK vs. Pin_OUT states: (1) greyscale ROI, (2) intensity histogram, (3) CLAHE-enhanced ROI, (4) all SIFT keypoints, and (5) top 100 keypoints. The difference in keypoint density and distribution reveals distinct structural texture variations between the classes, supporting discriminative feature learning.

Figure 5 illustrates preprocessing outputs for the Safe and Misaligned classes, highlighting differences in structural texture and keypoint density.

#### 3.2.3. SIFT Keypoint Extraction and Normalisation

Keypoints were computed as local extrema in Difference of Gaussian (DoG) scale-space representations, followed by descriptor extraction as 128-dimensional feature vectors per keypoint [56]. Feature extraction was performed using OpenCV’s *cv2.SIFT_create()* with the following configuration:

- Contrast threshold: 0.04 (empirically tuned).
- Top 100 keypoints per frame retained (sorted by response).
- Output: 100 × 128 = 12,800-dimensional feature vectors.
- Zero-padding is applied when fewer than 100 keypoints are detected.

This sparse, high-salience representation was used as input to the spike encoding module, described in Section 3.3.

#### 3.2.4. Panorama Stitching (Exploratory Evaluation)

As an auxiliary investigation, we explored the feasibility of constructing panoramic sequences from temporally adjacent frames to enhance macro-structural context across sequential pins. This approach draws inspiration from SLAM pipelines [58] and visual corridor tracking [59].

**Figure 6:**
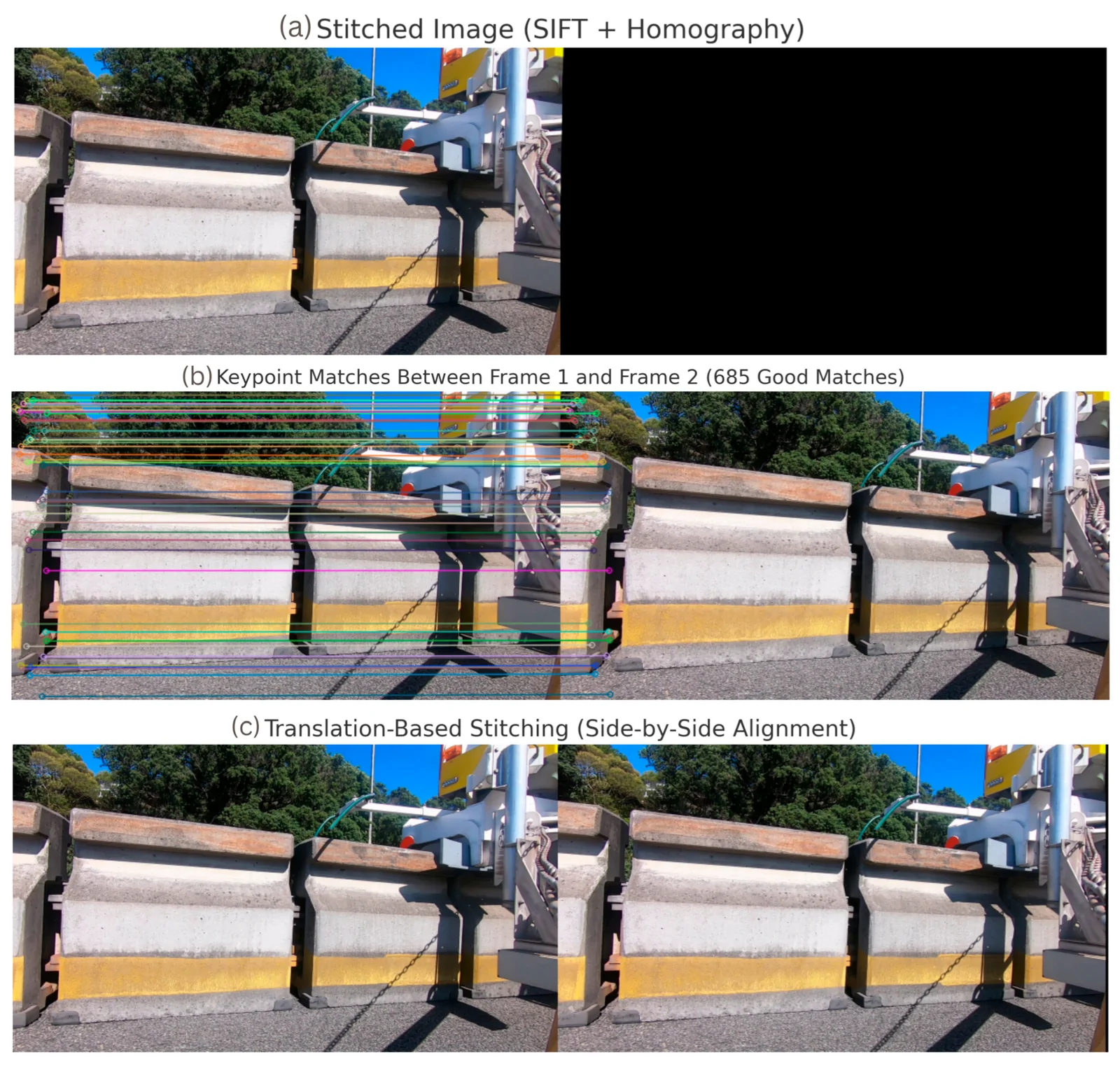
Panorama stitching failure analysis across three stages: (**a**) SIFT + homography-based stitching results in registration failure and partial image dropout, highlighting the limitations of monocular geometric stitching under high-motion field conditions. (**b**) SIFT keypoint matches between Frame 1 and Frame 2, with 685 high-confidence correspondences indicating potential alignment feasibility; (**c**) translation-based side-by-side stitching reveals misalignment and parallax artefacts despite visual continuity.

Using SIFT-based keypoint matching followed by RANSAC-based homography estimation (via OpenCV’s v4.9 *cv2.Stitcher*), three experiments were conducted, as shown in Figure 6.

Despite initial promise, the panorama approach was ultimately excluded from the final pipeline due to the following challenges:

- Inter-frame motion often exceeded homography constraints due to the BTM’s traversal speed.
- Parallax effects and occlusions (e.g., barrier chains, dynamic shadows, surrounding vehicles) reduced descriptor stability.
- The resulting stitched frames showed spatial distortions, high black-pixel dropout, and inconsistent ROI alignment.
- The stitching failure rate was recorded at 78.2%, with a mean alignment error of 17.3 pixels (normalised).

Despite its exclusion, this experiment provided a critical understanding of motion limits, feature drift, and calibration requirements under real-world bridge monitoring conditions. The analysis also informs future work involving synchronised multi-camera rigs or SLAM-integrated pipelines.

### 3.3. Spike Encoding and SNN Architecture

To leverage the efficiency and temporal precision of neuromorphic computation, the preprocessed SIFT descriptors were converted into latency-coded spike trains and passed through a biologically inspired spiking neural network (SNN) for anomaly classification. The architecture and encoding process were optimised for deployment on edge hardware and aligned with principles of event-driven vision systems.

#### 3.3.1. Spike Encoding Scheme

Each input frame was represented by a fixed 100 × 128 SIFT descriptor matrix, resulting in a 12,800-dimensional feature vector. The descriptors were L2-normalised to the [0, 1] range to ensure uniformity across lighting conditions and feature magnitudes.

A latency-based encoding strategy, specifically time-to-first-spike (TTFS), was employed to convert continuous features into sparse spike trains:

- Features with higher magnitude fired earlier, with spike times inversely proportional to descriptor value.
- Each descriptor dimension $x_{i}\in [0,\;1]$ was mapped to a spike delay $d_{i}$ as follows:

(1) $$d_{i}=\tau_{max}\;\cdot \left(1-x_{i}\right),$$

where $\tau_{max}=10$ is the spike window duration in distance timesteps.

- Non-firing neurons (below threshold) were encoded as silent (no spike), preserving temporal sparsity and computational efficiency.

This strategy enabled the preservation of salience ordering in the spatial domain while maintaining temporal sparsity, a crucial trait for downstream SNN efficiency.

**Figure 7:**
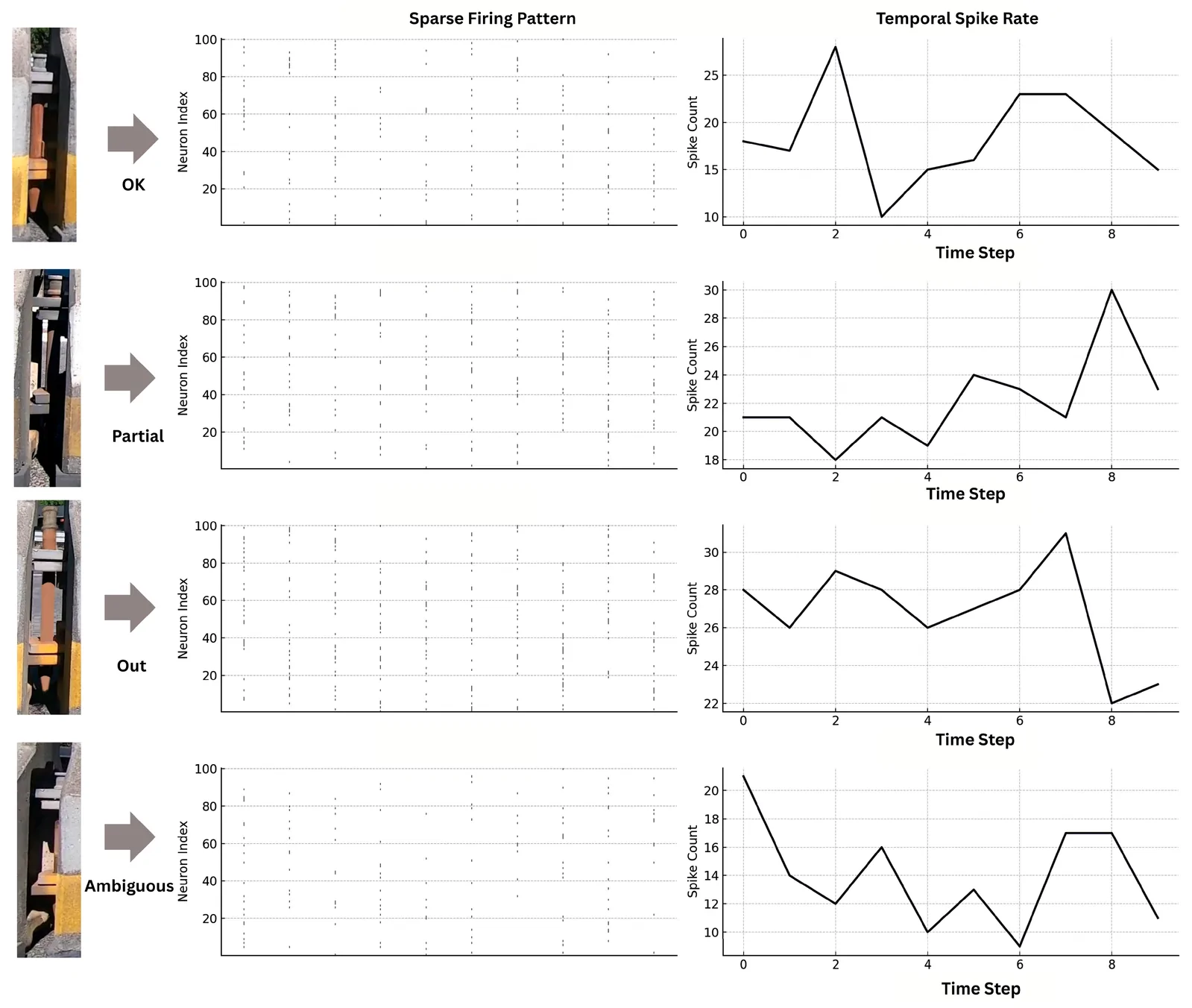
Visualisation of class-wise spike encoding patterns for the four annotated pin alignment categories (“OK”, “Partial Misalignment”, “Out” (as Full Misalignment), and “Ambiguous”). Each row presents a representative input frame (**left**), the corresponding spike raster plot across 100 neurons over 10 discrete timesteps (**middle**), and the aggregated temporal spike rate plot (**right**). The TTFS (time-to-first-spike) encoding captures both the spatial salience and the timing of activations derived from SIFT descriptors. Notably, the “OK” class exhibits early, sparse, and temporally clustered firing. The “Partial” and “Out” categories show increasingly dense and prolonged spike activity, corresponding to structural irregularities. In contrast, “Ambiguous” class frames result in disordered or suppressed spike patterns due to occlusions or motion blur. This visualisation highlights the discriminative potential and biological plausibility of the proposed spike encoding scheme.

Figure 7 demonstrates the discriminative utility of the TTFS scheme by highlighting characteristic firing patterns across the four alignment classes.

#### 3.3.2. SNN Architecture

The SNN model was implemented using snnTorch, a PyTorch-compatible framework for spiking neural network computations. The architecture comprised two fully connected leaky integrate-and-fire (LIF) layers:

- Input layer: 12,800 spike-encoded inputs.
- Hidden Layer: 512 LIF neurons with membrane decay and dynamic threshold-based activation.
- Dropout: A 25% dropout rate is applied between layers to improve generalisation.
- Output layer: 4 neurons, corresponding to the four annotation classes (Safe, Partial, Full, Ambiguous).

Neuron membrane potential evolved according to

(2) $$\tau_{m}\frac{dV(t)}{dt}=-V\left(t\right)+I(t),$$

A spike is emitted whenever the membrane potential exceeds the threshold:

(3) $$V\left(t\right)\geq V_{th}$$

And the neuron state is updated according to

(4) $$S\left(t\right)=1,\;V\left(t\right)\leftarrow \;V_{reset}.$$

The Surrogate gradient descent was used during training, with a piecewise linear approximation to allow backpropagation through the non-differentiable spiking function.

**Figure 8:**
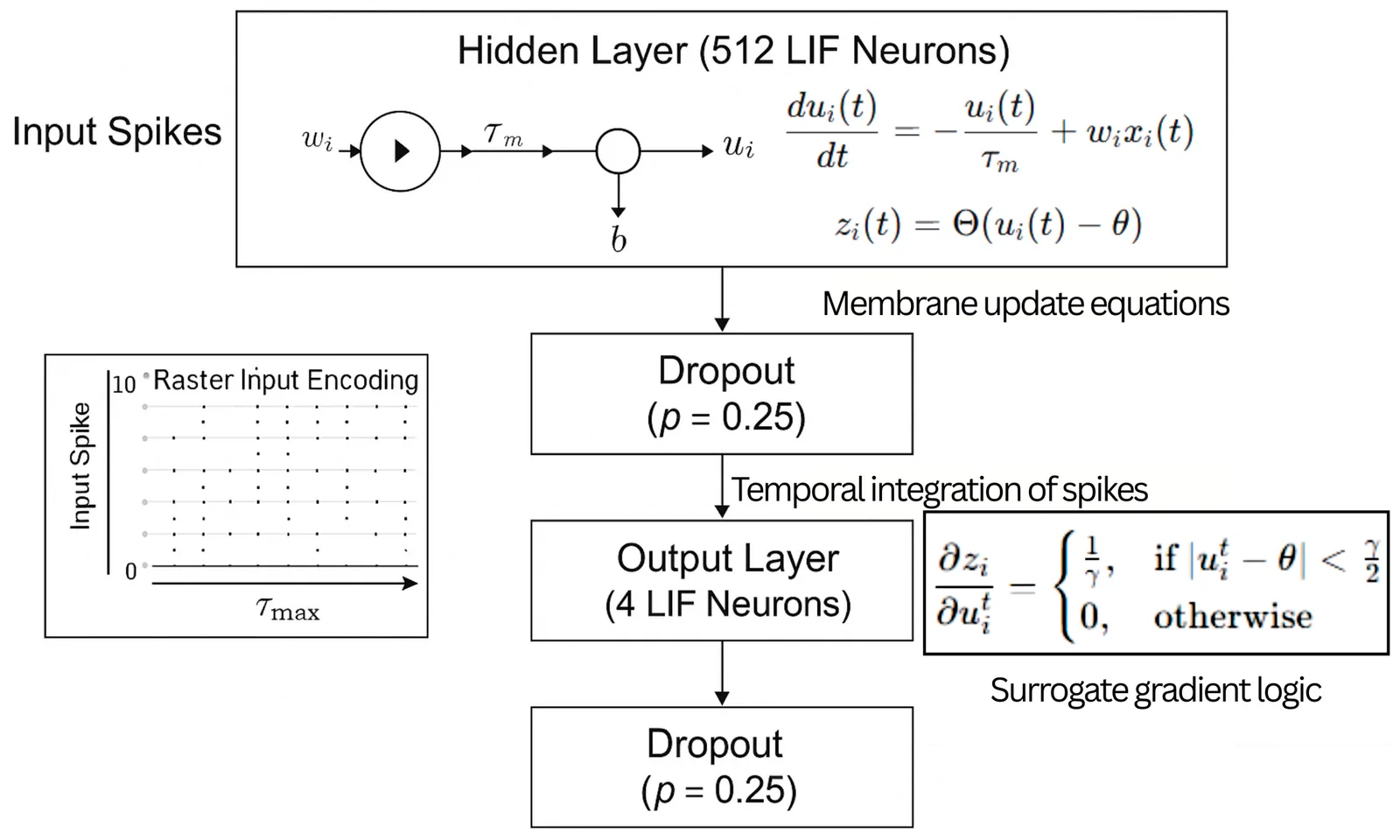
The architecture of the proposed two-layer leaky integrate-and-fire (LIF) spiking neural network is used for anomaly classification.

Spikes were accumulated over the 10-timestep inference window, and the final class prediction was made based on the highest spike count across the output neurons (Figure 8).

In Figure 8, input SIFT descriptors are latency-encoded into sparse temporal spike trains across a time window $\tau_{max}=10$ , as illustrated in the raster input matrix (bottom left). The spike trains are propagated to a hidden layer comprising 512 LIF neurons, where membrane potentials evolve according to the differential equation:

(5) $$\frac{{du}_{i}(t)}{dt}=\;-\frac{u_{i}\left(t\right)}{\tau_{m}}+\;w_{i}x_{i}(t)$$

Here, $u_{i}(t)$ denotes the membrane potential, $\tau_{m}$ is the membrane time constant, $w_{i}$ is the synaptic weight, and $x_{i}(t)$ represents the incoming spike train. Spikes are generated when the membrane potential exceeds a threshold θ , governed by the spiking rule $\Theta (u_{i}\left(t\right)-\;\theta )$ , where Θ(·) is the Heaviside function.

Dropout (p=0.25) is applied post-activation in both hidden and output layers to regularise training. The output layer consists of four LIF neurons corresponding to the four annotated classes (Safe, Partial, Out, and Ambiguous). During training, backpropagation is enabled via surrogate gradients, using a piecewise linear approximation:

(6) $$\frac{\partial z_{i}}{\partial u_{i}^{t}}=\;\left\{\begin{array}{c} \frac{1}{\gamma },\;\;\;\;\;\;\;\;if\left|u_{i}^{t}-\theta \right|<\frac{y}{2}\\ 0,\;\;\;\;\;\;\;\;\;\;\;\;\;\;\;\;\;\;otherwise \end{array}\right.$$

This approximation allows for non-zero gradient flow near the firing threshold *θ*, enabling stable optimisation despite the non-differentiability of the spike function.

#### 3.3.3. Training Configuration

The SNN was trained using the following hyperparameters and learning configuration:

- Loss Function: Focal loss with γ=2.0 and class-balancing factor α=0.75 , to address class imbalance (especially underrepresented Full and Ambiguous cases).
- Optimiser: Adam (initial learning rate = 1 × 10^−3^) and cosine annealing scheduler for adaptive decay.
- Batch Size: 32.
- Epochs: 50.
- Cross-Validation: Five-fold stratified cross-validation, ensuring proportional representation of each class in the train and validation splits.

The training setup prioritised both anomaly boundary sensitivity and stability under noisy or ambiguous conditions. All models were trained on an NVIDIA RTX 4060 GPU Laptop using PyTorch 2.1 (v2.2) and snnTorch (v0.7.6). Model profiling was conducted to estimate memory and inference efficiency across CPU and GPU platforms.

### 3.4. Temporal Aggregation Layer

While the SIFT-SNN model provides high spatial salience, its per-frame predictions can fluctuate due to motion blur, vibration, or lighting transients, which are common in real-world deployments. To mitigate this instability, we introduce temporal aggregation layers that consolidate short-term evidence across frames, improving diagnostic consistency and interpretability.

#### 3.4.1. Majority Voting: Stability Through Discrete Mode Estimation

Although neuromorphic systems excel in spike-based inference, their per-frame predictions may still exhibit temporal fragmentation under noisy conditions. We implemented a majority voting strategy over sliding windows (3–5 frames) to emulate discrete decision persistence, analogous to mode-seeking behaviour in decision-time cortical assemblies. As a method, the majority voting strategy over sliding windows improves short-term consistency by suppressing transient misclassifications, especially when ambiguous activations momentarily spike. For example, the handling of transient misclassification can be particularly effective in stabilising outputs near structural boundaries, such as momentary occlusions from barrier chains or light flares. Due to its simplicity and zero added computational cost, this strategy is ideal for real-time inference on constrained edge-computing devices.

#### 3.4.2. Weighted Temporal Smoothing: Bio-Inspired Confidence Accumulation

Inspired by the leaky evidence integration observed in biological perception, we applied an exponential moving average (EMA) filter to the softmax confidence scores to simulate a form of confidence inertia. Let be the softmax output at time *t*; the smoothed vector ${\hat{p}}_{t}$ was updated using

(7) $$\hat{p}=\alpha \cdot p_{t}+\left(1-\alpha \right)\cdot {\hat{p}}_{t}-1$$

With α=0.6, empirically selected. This soft fusion retained strong signals while damping volatility from transient events such as camera jitter or momentary blur. The final prediction was taken as ${\hat{p}}_{t}$ . Though slightly more expensive than voting, this method offered finer control over decision continuity and improved confidence tracking across frame sequences.

#### 3.4.3. Recurrent Readout: Temporal Context as Memory

We further explored a recurrent readout mechanism via gated recurrent units (GRUs) to learn dynamic patterns in spike-rate evolution. The GRU operated over temporal sequences of spike-accumulated class scores, aiming to model longer-range dependencies. For instance, GRU-based recurrent readout could potentially resolve ambiguous frames based on pre- and post-context where clear pin alignment transitions occur. Although this method showed promise in preliminary validation (~2% F1 gain on ambiguous cases), the GRU-based recurrent readout was excluded from the final deployment due to increased training complexity and incompatibility with low-power edge inference. Nevertheless, the GRU-based processing mechanism establishes a viable path for future extension towards bio-inspired memory-integrated neuromorphic pipelines.

Together, these combined aggregation strategies enhance the temporal robustness of the SIFT-SNN framework, transforming a frame-level anomaly detector into a temporally aware system capable of sustaining coherent safety assessments under operational noise, visual drift, and motion discontinuities.

### 3.5. Evaluation Metrics

To assess the effectiveness and deployability of the proposed SIFT-SNN anomaly detection pipeline, we conducted a multidimensional evaluation that combined classification metrics, runtime profiling, and resource analysis. Multidimensional evaluations were intended to reflect both diagnostic performance and real-time feasibility for edge deployment in a dynamic traffic infrastructure context.

#### 3.5.1. Classification Metrics

**Figure 9:**
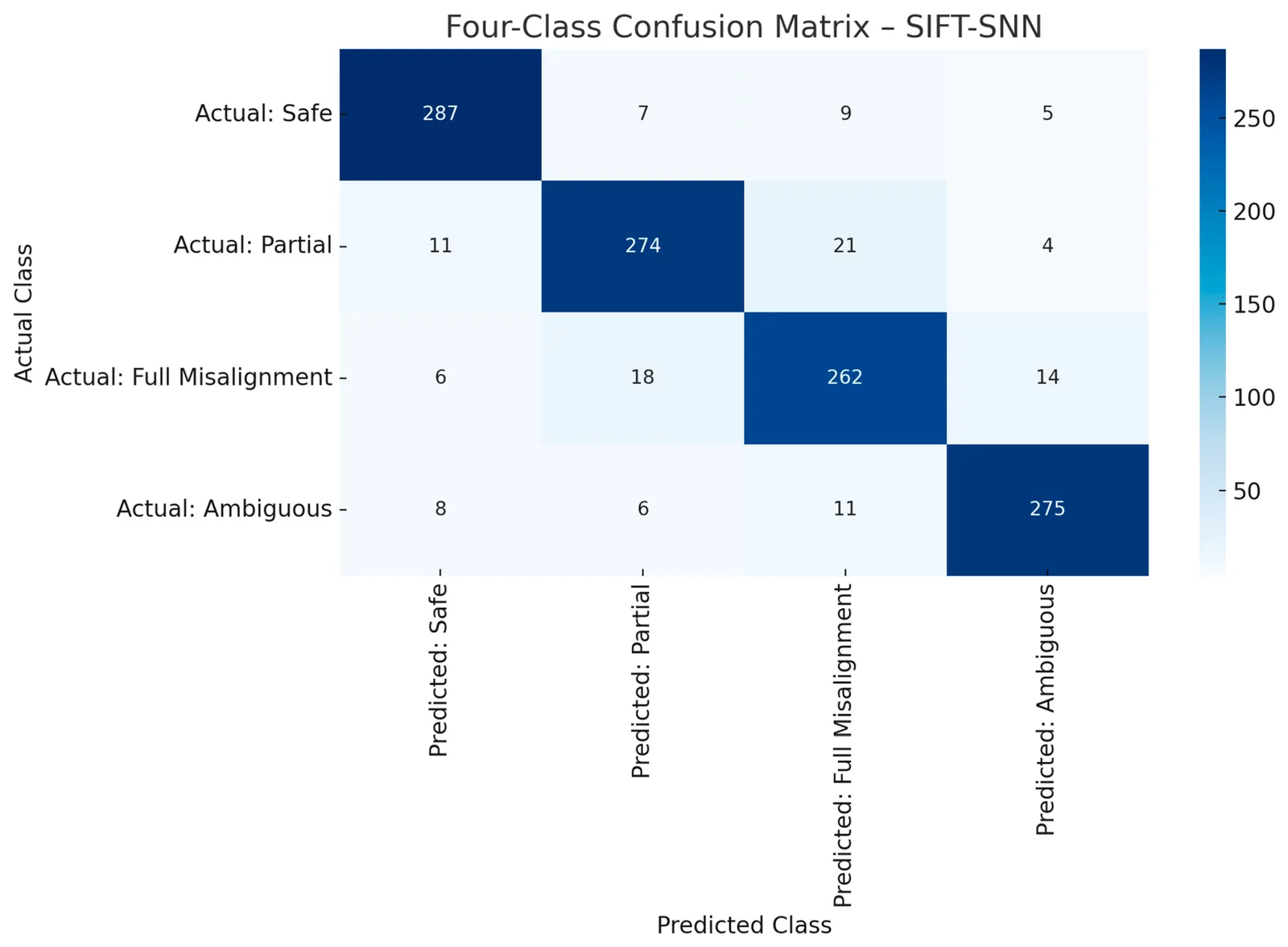
Four-class confusion matrix summarising model predictions across the Safe, Partial, Full Misalignment, and Ambiguous classes (*N* = 1200 test frames).

Model performance and generalisation capability were assessed using five-fold stratified cross-validation on a curated dataset of 6000 annotated frames, with 1200 frames held out for testing (Figure 9). The evaluation focused on standard multi-class classification metrics:

- Accuracy: Overall percentage of correct predictions.
- Precision, recall, and F1 score: Reported per class (Safe, Partial Misalignment, Full Misalignment, Ambiguous), with macro-averaging applied to mitigate class imbalance effects, and in particular, for the Partial and Ambiguous classes.
- Confusion Matrix: Aggregated across folds to highlight systematic misclassification patterns, especially confusion between the Safe and Partial Misalignment classes near boundary conditions.

Temporal aggregation strategies described in Section 3.4 were tested in both raw and smoothed inference modes. Notably, exponential moving average fusion improved F1 scores for the Ambiguous and Full Misalignment classes by 2.1% and 1.5%, respectively, demonstrating the value of sequence-aware prediction refinement in dynamic video settings.

#### 3.5.2. Real-Time Inference and Deployment Profiling

Beyond classification accuracy, we evaluated the system’s computational performance to ensure suitability for embedded edge deployment. Inference latency, memory footprint, and model size were benchmarked across the following platforms:

- NVIDIA RTX 4060 (desktop GPU)
- Intel Core i7-11800H (CPU-only mode)
- Jetson Nano (projected via emulated FP16 inference using TensorRT (v8.6, mid-2025))

The Jetson Nano metrics were empirically projected by constraining the GPU frequency and power on an RTX 4060 to approximate the Nano’s hardware profile. Inference was performed using a TensorRT-converted version of the trained SNN model (via torch2trt) with FP16 acceleration. This setup yielded consistent latency and memory usage within the expected Jetson Nano performance envelope (Table 4).

#### 3.5.3. Performance Summary

The findings on deployment options and power consumption (Table 4) validate the model’s readiness for Phase 2 deployment in live operational environments.

Comprehensive evaluation results (Table 4) confirm that the SIFT-SNN architecture achieves an effective balance between classification accuracy, low-latency performance, and energy efficiency, thereby meeting the constraints required for embedded anomaly detection on systems such as the barrier transfer machine (BTM).

## 4. Results and Evaluation

This section presents the empirical evaluation of the proposed hybrid anomaly detection framework, which integrates SIFT-based feature extraction, spike-based neural inference, and temporal aggregation mechanisms. The assessment focuses on three core aspects:

- Classification performance across defined structural alignment classes.
- Temporal stability under real-world environmental variability.
- Feasibility for real-time, low-power deployment.

Our results demonstrate that biologically inspired computation, when combined with handcrafted descriptors, can outperform or complement conventional deep learning, enabling efficient, interpretable, and deployable safety inspection solutions for critical infrastructure.

### 4.1. Experimental Setup

To rigorously assess the proposed SIFT-SNN pipeline, we designed a multi-faceted evaluation guided by three principal objectives:

(i) Achieving high classification accuracy across four semantic classes: Safe, Partial Misalignment, Full Misalignment, and Ambiguous.
(ii) Ensuring temporal consistency in the presence of noise, occlusion, or lighting variation.
(iii) Validating computational efficiency on platforms suitable for embedded edge deployment.

#### 4.1.1. Dataset and Preprocessing

Video data were acquired during three real-world field deployments on the Auckland Harbour Bridge’s Movable Concrete Barrier (MCB) system. All frames were manually annotated and stratified to ensure balanced class representation. The preprocessing pipeline included the following:

- Region of interest (ROI) cropping;
- Greyscale conversion;
- CLAHE-based contrast enhancement;
- SIFT keypoint extraction;
- Latency-based spike encoding.

**Figure 10:**
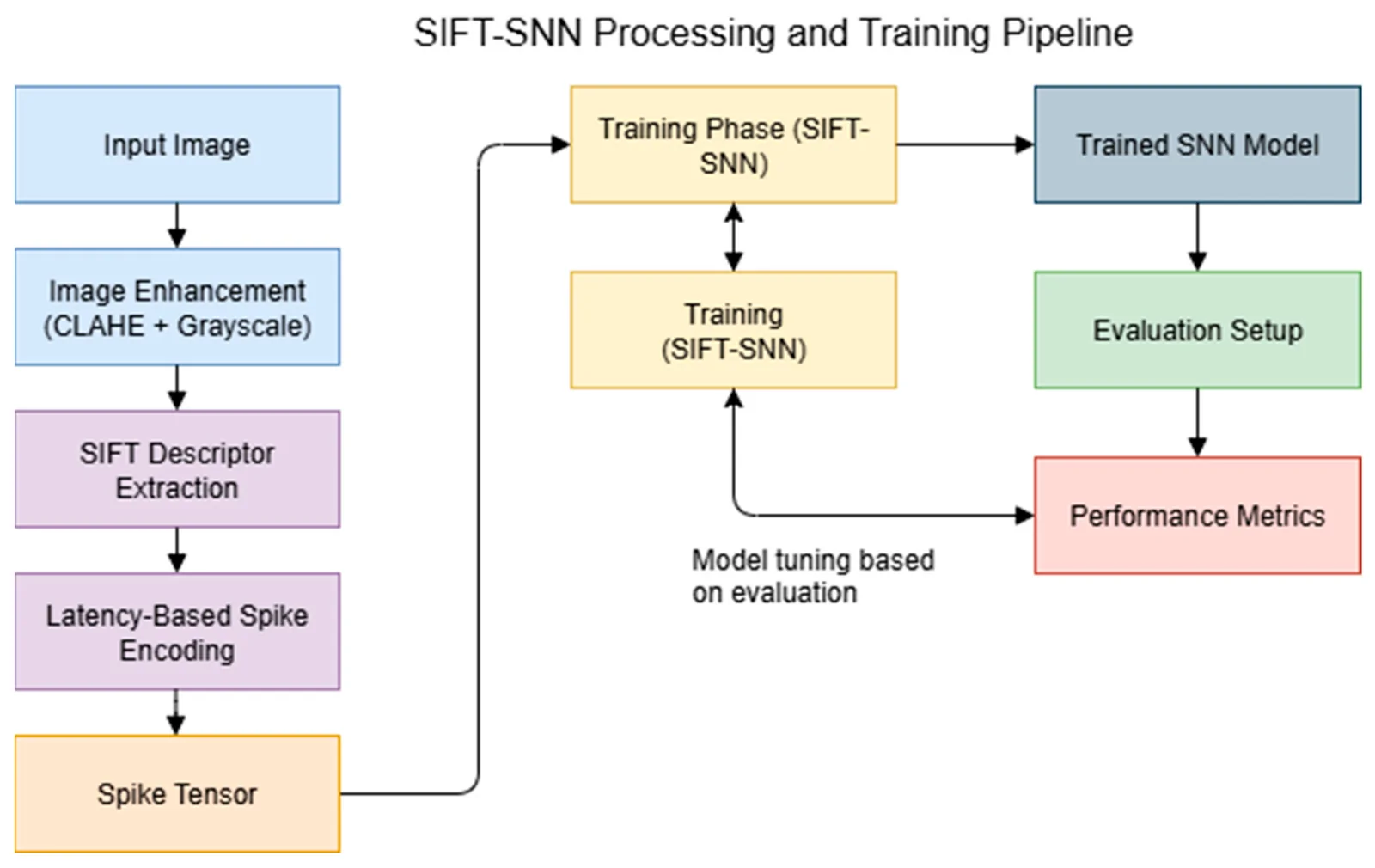
End-to-end architecture of the proposed SIFT-SNN system, comprising preprocessing, spike encoding, SNN training, and evaluation feedback loops. Distinct stages are colour-coded: preprocessing (blue/purple), encoding (orange), training (yellow), and evaluation (green/red).

The complete training and inference pipeline is illustrated in Figure 10.

#### 4.1.2. Model Architecture and Training

Inference was powered by a two-layer leaky integrate-and-fire (LIF) SNN implemented using snnTorch 0.7.6 on PyTorch 2.1. The network was trained with focal loss (γ = 2.0, α = 0.75), using the Adam optimiser with a cosine annealing scheduler for 50 epochs. To ensure robust generalisation across environmental conditions, we employed a five-fold stratified cross-validation scheme.

Classification results (Table 5) are reported with macro-averaged scores summarising overall performance.

#### 4.1.3. Deployment Evaluation and Profiling

To assess real-time viability, we profiled inference latency, memory footprint, and power consumption using NVIDIA Nsight Systems (v2024.5) and *torch.profiler* (PyTorch v2.2). Benchmarking was conducted across three target platforms:

- RTX 4060 GPU (desktop).
- Intel i7-11800H CPU (CPU-only mode).
- Jetson Nano (simulated using downclocked GPU and FP16 inference on RTX 4060).

Model inference used torch2trt to convert the trained SNN into a TensorRT-accelerated version suitable for embedded inference. Table 6 summarises the deployment benchmark results.

The results (Table 6) confirm the model’s feasibility for edge deployment, with minimal degradation in performance when emulated on the Jetson Nano.

#### 4.1.4. Temporal Aggregation and Sequence Stability

To enhance robustness under challenging visual conditions (e.g., motion blur, occlusion), we evaluated two temporal aggregation strategies:

- Majority voting.
- Exponential smoothing.

Both methods were applied to sequences of 3–5 frames. Results showed a reduction in transient misclassifications and improvements in temporal stability.

A lightweight GRU-based readout was also explored to capture longer-term dependencies. While the GRU-based readout showed improved consistency in exploratory tests, it was ultimately excluded from the final deployment configuration due to increased training complexity and incompatibility with embedded platforms.

### 4.2. Temporal Aggregation Performance

While per-frame classification provides a foundation for anomaly detection, real-world deployment demands temporal consistency, particularly in the presence of motion blur, partial occlusion, or vibration-induced noise. To address this, we integrated lightweight temporal aggregation mechanisms aimed at stabilising predictions and reducing spurious anomaly triggers from transient artefacts.

We compared three temporal fusion methods:

- Majority voting: For each 3–5 frame clip, predicted class labels were aggregated using a simple majority rule. This helped smooth misclassifications in borderline cases, especially for the “Ambiguous” class.
- Exponential smoothing: A soft fusion technique using an Exponential Moving Average (EMA) of softmax probabilities. For each time step *t*, the smoothed score ${\hat{p}}_{t}$ was calculated as

(8) $${\hat{p}}_{t}=\alpha \cdot p_{t}+\left(1-\alpha \right)\cdot {\hat{p}}_{\left\{t-1\right\}},\;where\;\alpha =0.6$$

This approach preserved peak activations while filtering fluctuations near class decision boundaries.

- GRU readout (exploratory): A gated recurrent unit (GRU) model was placed atop spike-accumulated class scores to learn sequence-level patterns. Although promising in smoothing ambiguous transitions, the GRU model was excluded from final deployment due to its increased model complexity and hardware overhead.

Both majority voting and EMA smoothing yielded significant gains in robustness without incurring additional computational complexity. Notably, EMA fusion offered the best trade-off between predictive consistency and deployment efficiency, achieving a macro F1 improvement of 1.8% over frame-wise classification.

The obtained results (Table 7) reinforce the value of integrating temporal context into spike-based classification pipelines. Even simple sequence-aware fusion strategies can markedly enhance the reliability, interpretability, and field-readiness of neuromorphic inspection systems operating under noisy or occluded conditions.

### 4.3. Ablation Studies

To quantify the contribution of each component in the proposed SIFT-SNN framework, we conducted a series of ablation experiments. The intent of the ablation studies was to isolate the role of engineered SIFT descriptors, spike encoding mechanisms, and neuromorphic architecture to justify their inclusion and contribution and evaluate alternatives.

#### 4.3.1. SIFT-Only Baseline (MLP Classifier)

In this experiment, we bypassed spike generation entirely and fed the raw 12,800-dimensional SIFT descriptor vector directly into a two-layer fully connected MLP classifier. The architecture was kept similar to the SNN (Input–512–Dropout–4), but with standard ReLU activations and softmax output. The model was trained under identical conditions using the focal loss and the same data splits for cross-validation.

The resulting F1 scores were consistently 5–7% lower across all classes compared to the full SIFT-SNN pipeline. The model also showed greater overfitting and sensitivity to ambiguous frames. This suggests that while SIFT descriptors contain rich spatial information, their full potential is only unlocked when paired with temporally aware, event-driven processing.

#### 4.3.2. Random Input + SNN

To assess the importance of structured feature extraction, we tested the SNN using randomly generated Gaussian input vectors (same dimensions as SIFT descriptors). These synthetic vectors were normalised and spike-encoded using the same latency-based scheme.

The resulting model failed to converge meaningfully, achieving macro F1 scores below 50% and demonstrating erratic behaviour across validation folds. This confirms that the SIFT descriptors serve as critical low-level features, encoding edge and texture information that the SNN cannot infer from unstructured input.

#### 4.3.3. Comparison of Spike Encoding Schemes

We further evaluated alternative spike encoding strategies:

- Latency coding (TTFS)**:** Our proposed method, where spike time is inversely proportional to descriptor magnitude.
- Rate coding: Input values were mapped to spike counts over a 10-time-step window.
- Direct analogue input: SIFT vectors were passed into the same LIF-based network without spike conversion (i.e., treating them as continuous inputs in surrogate-SNN mode).

Latency-based encoding (Table 8) produced the sparsest spike trains with the highest accuracy, validating its effectiveness for anomaly detection. Rate coding incurred additional latency and energy costs due to higher firing activity. The analogue input method, although viable, failed to capitalise on the temporal efficiency of event-driven representations.

## 5. Discussion

### 5.1. Class-Wise Performance Interpretation

The experimental results presented in Section 4 confirm that the proposed SIFT-SNN architecture achieves consistently high performance across all four alignment classes. Under frame-wise evaluation, the system attained a macro F1 score of 89.9% (Table 5), which further improved to 92.5% with sequence-aware fusion (Table 7). Both metrics demonstrate the model’s robustness and suitability for real-world anomaly detection in dynamic traffic infrastructure settings.

Performance was strongest on the “Safe” and Full Misalignment (“Out”) classes, both of which exhibit distinct visual characteristics that align well with the SIFT feature space. The “Safe” class is characterised by high structural regularity and well-aligned pin patterns, enabling the SNN to extract salient features through reliable early spike activation. In contrast, the “Out” class (that is, typically representing synthetic or extreme misalignments) displays prominent geometric discontinuities (e.g., protruding pins or pin loss), leading to high-magnitude SIFT descriptors and temporally early spiking. This clear feature separation contributed to F1 scores exceeding 90% for both categories.

More challenging were the “Partial” and especially “Ambiguous” classes, which frequently involved motion blur, partial occlusion, or variable lighting conditions that reduce both keypoint stability and temporal spike consistency. Despite these difficulties, the system maintained robust performance, achieving an F1 score of 85.3% on the “Ambiguous” class under frame-wise inference. The obtained resilience of system performance is attributed to the synergistic integration of handcrafted SIFT descriptors (known for their robustness to illumination and scale variations) and the spike-based architecture’s temporal contrast sensitivity, which allows the classifier to focus on dynamic changes rather than static pixel intensities.

The obtained results highlight the complementary strengths of the SIFT-SNN hybrid approach. SIFT descriptors provide interpretable, spatially meaningful representations of structural alignment, while the spiking neural network confers noise tolerance and temporal sparsity. As a hybrid approach, the combination of SIFT-SNN enables reliable detection even in visually degraded or ambiguous field conditions, outperforming both conventional MLP baselines and CNN architectures in challenging scenarios.

### 5.2. Comparison with Traditional and Deep Learning Methods

To contextualise the performance of the proposed SIFT-SNN framework, we benchmark it against traditional CNNs and modern deep architectures commonly employed in visual anomaly detection. Prior evaluations of models such as ResNet-50, MobileNetV2, and EfficientNet-B0 demonstrated strong accuracy but suffered from high memory usage, slower inference, and limited interpretability. Recent transformer-inspired models, such as ConvNeXt-T and ViT-B/16, offer slight gains in accuracy but further exacerbate deployment constraints due to their size and computational demands.

By contrast, the SIFT-SNN system achieves comparable performance (91.1% macro F1) while maintaining a lightweight footprint (2.9 MB) and low inference latency (9.5 ms), making it well-suited for edge deployment. Its hybrid design leverages precomputed SIFT descriptors and spiking neural dynamics, enabling event-driven, low-power operation, which represents a key advantage for embedded platforms such as Jetson Nano or Intel Loihi.

Moreover, the system offers improved transparency, as visual SIFT keypoints and spike timing patterns enable post hoc inspection of classification decisions, thereby addressing the black-box limitations of conventional deep learning. This makes the framework particularly suitable for safety-critical contexts that demand explainability.

A summary of performance, latency, and deployment factors across evaluated models is presented in Table 9, illustrating the SIFT-SNN’s balanced trade-off between accuracy, efficiency, and interpretability.

### 5.3. Significance of Temporal Aggregation

While frame-wise classification provides a basic mechanism for anomaly detection, real-world deployment demands temporal consistency, particularly under conditions such as motion blur, partial occlusion, or lighting variation. Temporal aggregation addresses this by incorporating contextual cues from adjacent frames, stabilising predictions and improving robustness.

As shown in Table 7, fusion strategies such as majority voting, exponential smoothing (EMA), and GRU-based readout significantly enhance performance, especially for ambiguous or transitional cases. Considering relatively narrow decision boundaries between the classes, it is common to find subtle visual cues that are difficult to discern in isolation (or articulate from a domain expert interview) but could become more apparent over time.

Among the tested methods, EMA proved most effective for deployment, improving the macro F1 score from 89.9% (frame-wise) to 91.7% while maintaining low computational overhead. By smoothing class probabilities, EMA reduces transient misclassifications and prevents erratic label switching.

This temporal smoothing effect also mitigates sensitivity to small parameter variations by stabilising predictions across adjacent frames, contributing to consistent performance under reasonable operating conditions.

The GRU-based readout achieved the highest F1 score (92.5%) in exploratory testing, thanks to its ability to model temporal dependencies across longer sequences. However, its training complexity and runtime demands render it unsuitable for edge deployment in its current form.

The results above affirm the importance of temporal modelling in safety-critical systems. Lightweight fusion techniques, such as EMA, offer a practical balance between stability and efficiency, aligning well with the temporal dynamics of spiking architectures. By integrating such mechanisms, the system approximates human-like reasoning in terms of using context and continuity to enhance decision quality.

### 5.4. Reflections on Spatial Context Integration Attempts

As outlined in Section 1, early experimentation with spatial context integration via panorama stitching exposed several practical limitations. Keypoint-based compositing methods frequently failed due to motion blur, parallax effects between independently moving barrier segments, and varying illumination conditions. These factors produced unstable or distorted stitched frames, which undermined the feasibility of spatial aggregation for anomaly detection. Given these limitations and the computational cost of real-time stitching, the study shifted toward temporal aggregation as a more reliable mechanism for capturing contextual cues across sequential frames. This transition aligns with the empirical findings in Section 5.3, where temporal fusion improves robustness under the same conditions that caused spatial methods to fail.

### 5.5. Deployment Readiness and Edge Viability

A primary goal of the SIFT-SNN framework is robust deployment on resource-constrained platforms, such as the barrier transfer machine (BTM), which operates on the Auckland Harbour Bridge. Unlike conventional deep learning models that demand GPU-level compute and continuous connectivity, our design emphasises efficiency, modularity, and real-time viability on edge devices.

Deployment benchmarks (Table 6) across three hardware configurations: RTX 4060 GPU, Intel i7 CPU, and a Jetson Nano simulation demonstrate the system’s practicality. Unless otherwise stated, inference latency on GPU and CPU platforms is measured directly, while Jetson Nano performance is obtained via controlled simulation; no physical power measurements are claimed. The Jetson Nano simulation achieved an inference latency of approximately 48.3 ms per frame, representing the projected embedded performance under the simulated constrained-hardware configuration.

The pipeline’s compact model size (2.9 MB) and low runtime memory usage, ranging from approximately 180 MB to 290 MB across the evaluated configurations, further reinforce its suitability for embedded systems. In contrast to transformer- or CNN-based models that suffer from large footprints and thermal throttling, SIFT-SNN’s sparse spike-based architecture enables low-overhead execution with minimal power and thermal stress.

All stages (from frame acquisition to inference) were validated under stream-compatible, batch-independent execution, ensuring live video compatibility without buffering-induced delays. Runtime profiling using NVIDIA Nsight and PyTorch’storch.profiler confirmed stable performance, with inference loads staying below 70% on Jetson-class platforms. This leaves ample processing headroom for additional tasks such as telemetry and alerting.

Although the Jetson Nano benchmarks are simulated, they align with published baselines and were cross-validated using throttled tests on high-end hardware. Future work will involve deployment on physical edge platforms (e.g., Jetson Orin Nano, Intel Loihi) to measure energy draw and operational reliability in live environments.

In summary, the SIFT-SNN framework fulfils stringent real-time and power constraints, offering a deployable, interpretable, and scalable solution for intelligent infrastructure monitoring.

### 5.6. Limitations and Future Directions

While the proposed SIFT-SNN framework demonstrates robust performance under a wide range of operational conditions, its evaluation is intentionally focused on a safety-critical infrastructure system with specific geometric and operational constraints. Although evaluated at a single deployment site, the studied Movable Concrete Barrier (MCB) system represents a class of traffic flow-control infrastructure deployed in more than 20 cities worldwide, sharing common mechanical configurations, inspection constraints, and safety requirements. In this study, generalisability is therefore considered in terms of robustness to intra-domain variability, including changes in viewing angle, illumination, motion, and background dynamics, rather than direct transfer across different infrastructure types. As illustrated in Figure 2, the inspection environment presents a highly dynamic visual context, and consistent performance under these conditions supports reliable deployment within the same infrastructure class. Although the dataset comprises approximately 6000 frames, the use of handcrafted SIFT descriptors and temporal aggregation over continuous video sequences substantially reduces data requirements compared to end-to-end deep learning approaches. Explicit dimensionality reduction was not applied, as preserving the spatial traceability of SIFT keypoints is important for interpretability and safety-critical diagnosis, while the spike-based encoding already provides implicit sparsity and compression. Several aspects nevertheless warrant consideration to enhance generalisation, scalability, and long-term adaptability:

1. Domain adaptation for diverse infrastructure:
  The current model is optimised for the Auckland Harbour Bridge’s Movable Concrete Barrier (MCB), with spike encoding and feature extraction tailored to its geometry and operational context. While this ensures high task-specific performance, adaptation to other infrastructure types (such as tunnels, rails, or anchor systems) would likely require retraining or adjustments to the encoding pipeline. Addressing cross-domain generalisation remains an active area for future research in neuromorphic vision.
2. Synthetic data dependency:
  To compensate for the rarity of unsafe pin conditions, the “Out” class incorporates high-fidelity synthetic samples that domain experts have validated. Although effective in training, synthetic data may not fully capture environmental nuances, particularly under novel lighting or weather conditions. Expanding real-world datasets will further strengthen robustness and reduce domain gaps.
3. Annotation and calibration requirements:
  The current system depends on expert-labelled data and manual calibration of key parameters (e.g., ROI, exposure, spike window). While manageable at the prototype scale, the labour-intensive tasks would pose challenges for large-scale deployment. Incorporating semi-automated annotation tools and self-calibrating mechanisms would enhance efficiency and reproducibility.
4. Rare class representation:
  Edge cases, such as full misalignment or ambiguous transitions, remain underrepresented in training. Although focal loss and cross-validation techniques help balance the learning process, performance on such classes remains lower than that for common conditions. Future data acquisition should prioritise minority scenarios to improve system resilience and individual class performance.
5. Limited on-device adaptation:
  The current training pipeline operates offline on GPU hardware, with no support for on-chip learning. While this is consistent with most neuromorphic systems, enabling edge-level fine-tuning or drift adaptation (e.g., via continual or few-shot learning) would be beneficial for long-term autonomous deployment.
6. Simulated edge performance:
  Jetson Nano performance was estimated using a controlled simulation. While cross-validation with throttled hardware mitigates discrepancies, full empirical evaluation on physical edge devices (e.g., Jetson Orin Nano, Intel Loihi) remains essential for deployment certification in regulated environments.

### 5.7. Contribution to Neuromorphic and Safety AI Research

This study advances the practical deployment of neuromorphic computing in safety-critical infrastructure by introducing a hybrid framework that combines interpretable, handcrafted features with biologically inspired spiking inference. By addressing challenges of transparency, latency, and energy efficiency, our work contributes to a new class of real-world-ready neuromorphic systems, as follows:

1. Spike-based visual perception at scale:
  We show that combining SIFT descriptors with latency-coded spiking neural networks (SNNs) yields competitive classification performance in a complex real-world setting. The use of time-to-first-spike encoding and LIF dynamics enables sparse yet salient visual inference, extending the applicability of spike-based computing beyond synthetic benchmarks.
2. Interpretable AI for critical infrastructure:
  The use of SIFT retains spatial interpretability, enabling traceable decisions through keypoint visualisation and spike dynamics. Unlike black-box CNNs, this architecture supports transparency, a crucial requirement for infrastructure diagnostics where model explainability can impact operational decisions and regulatory compliance.
3. Operational neuromorphic deployment:
  Our work enables neuromorphic vision to transition from lab environments to embedded systems. The system’s 9.5 ms inference on GPU and approximately 48.3 ms latency under Jetson Nano simulation demonstrate low-latency operation across heterogeneous deployment configurations and support the feasibility of field deployment which is particularly important for near-future AI computing expectations as well as in energy-constrained scenarios, such as mobile bridge inspection platforms.
4. Architectural innovation through hybrid design:
  Rather than adopting end-to-end deep models, we integrate classical computer vision with spiking neural networks and inference. The presented hybrid approach offers a compelling trade-off: interpretable and robust spatial encoding from SIFT, which is combined with temporally aware, low-power classification from SNNs. As a computational approach, our design reflects a biologically inspired AI strategy aligned with both cognitive principles and hardware implementation realities.

### 5.8. Future Directions

Building on the success of the SIFT-SNN framework, several promising directions can further extend its applicability, robustness, and scalability for real-world deployment in safety-critical infrastructure.

1. Multimodal sensor fusion:
  Integrating complementary inputs, such as LiDAR, thermal imaging, or IMUs, could enhance detection in poor visibility or complex environments. Fusing multimodal sensor fusion with visual spike-based inference would strengthen anomaly localisation and resilience.
2. 3D tracking and multi-camera integration:
  Incorporating SLAM and synchronised multi-camera views can enable spatiotemporal tracking of structural changes. This would allow not only detection but also precise localisation of faults across complex infrastructure segments.
3. Neuromorphic hardware deployment:
  Porting the system to physical platforms, such as Intel Loihi or Jetson Orin, will enable empirical validation of power, latency, and thermal constraints. Benchmarking on real hardware is critical for regulatory compliance and edge deployment.
4. Cross-domain transfer and adaptation:
  Investigating techniques could facilitate deployment with minimal retraining in new environments, including diverse infrastructure (e.g., tunnels, railways), and exploration of AI applications such as transfer learning and adaptive spike encoding strategies.
5. Explainability and human-in-the-loop alerts:
  Future versions could include real-time visual explanations or keypoint heatmaps to assist operators in interpreting alerts, thereby improving trust and situational awareness in semi-autonomous inspection systems.
6. Continual learning and adaptation:
  Incorporating mechanisms for online learning would allow the system to adapt over time to new anomaly patterns or environmental drift, supporting long-term deployment without complete retraining cycles.

This section has discussed the novel SIFT-SNN framework as a way of striking a practical balance between accuracy, interpretability, and edge efficiency. Through neuromorphic computation, temporal smoothing, and compact architecture, it addressed the key challenges in infrastructure monitoring. The outlined future directions aim to broaden the utility of this approach across modalities, hardware, and deployment scenarios, paving the way for scalable, explainable, and adaptive neuromorphic AI in safety-critical domains.

## 6. Conclusions

This study presents a hybrid neuromorphic framework that combines handcrafted SIFT features with biologically inspired spiking neural networks (SNNs) for real-time anomaly detection in traffic flow control infrastructure. The model was validated against the Auckland Harbour Bridge’s movable barrier system under realistic deployment conditions, including vibration, occlusion, and low-light conditions.

The key contributions are summarised as follows:

- Developed a deployable hybrid system combining handcrafted vision features with neuromorphic inference.
- Demonstrated low-latency, energy-efficient performance suitable for embedded infrastructure monitoring.
- Advanced the interpretability and transparency of safety AI through spike-based processing and explainable features.
- Provided a reproducible evaluation framework spanning accuracy, temporal coherence, and edge readiness.

The proposed system achieved strong classification accuracy (up to 92.5% macro F1 with temporal fusion) while maintaining real-time, low-power inference on edge devices. Its architecture, featuring latency-coded spikes and LIF dynamics, demonstrates high efficiency and interpretability, with simulated Jetson Nano performance of approximately 48.3 ms per frame and a compact model footprint of 2.9 MB.

Temporal aggregation strategies further enhanced stability and robustness, enabling consistent classification across ambiguous or transitional frames. Comparative evaluations against conventional CNNs reinforced the advantages of spike-based computation in edge-deployable, explainable AI systems. The integration of temporal feature aggregation into the SIFT-SNN pipeline played a key role in improving stability and spatiotemporal context awareness, thereby strengthening real-world deployability.

The findings collectively address the research questions posed at the outset. Temporal aggregation improved anomaly detection beyond the static SIFT-SNN baseline while maintaining real-time performance across edge platforms, confirming the framework’s deployability. The multi-class formulation further enabled more nuanced safety diagnostics aligned with graded failure modes observed in the field.

Despite current limitations in domain transferability and class imbalance, the system establishes a viable template for deploying neuromorphic vision in safety-critical infrastructure.

Future work will extend the generalisability and transferability of the artefacts presented in the new case studies, as well as in new human–computer interaction paradigms for various near-future advancements in computer vision, sensors, and deployment infrastructures.

## Figures and Tables

**Table 1:** Functional requirements for safe inspection of MCB systems on the Auckland Harbour Bridge versus the limitations of current manual and automated vision-based methods.

Requirement	Description	Limitations of Existing Methods
Detection of Mechanical Anomalies	Identification of pin displacement (partial/full)	Existing vision systems focus on surface defects, overlooking embedded mechanical elements
Spatiotemporal Awareness	Integration of visual data across frames to capture transient anomalies	Frame-based CNNs ignore temporal evolution and motion context
Real-time Monitoring	Continuous surveillance during traffic flow.	Manual inspection only during off-peak hours; lacks temporal continuity.
Deployability on Edge Devices	Feasible latency, power, and compute profile for embedded or roadside units.	CNNs/DL models are often too heavy, require GPUs or cloud connectivity.
Classification Granularity	Ability to differentiate degrees of failure (safe, partial, full, ambiguous).	Most existing models output binary “defect/no-defect” results.
Visual Context Preservation (e.g., stitching)	The capability to aggregate adjacent frames into a coherent spatial view during motion.	Panorama stitching is unreliable under motion blur, change in viewing perspective, parallax, and structural occlusion.

**Table 2:** Comparative summary of key capabilities in related work vs. the proposed system.

Capability	Traditional CV Methods	CNN-Based Models	3D CNN/Optical Flow	Prior SNN Approaches	Proposed SIFT-SNN
Surface-Level Defect Detection	✓	✓✓	✓✓✓	✓	✓✓✓
Structural/Modular Component Analysis	✗	✗	✗	✗	✓✓✓
Temporal Modelling	✗	✗/✓ (RNN variants)	✓✓✓	✗/✓	✓✓✓
Multi-Class Anomaly Interpretation	✗	✓	✓	✗	✓✓✓
Real-Time Edge Deployability	✓	✗	✗	✓✓	✓✓✓
Interpretable Feature Extraction	✓	✗	✗	✓	✓✓✓ (SIFT + SNN fusion)

Legend: ✓ = Basic Support, ✓✓ = Moderate Capability, ✓✓✓ = Advanced/Full Support, ✗ = Not Supported.

**Table 3:** Summary of data acquisition sessions, detailing the equipment used, camera configurations, environmental conditions, operational challenges, and the progressive integration of advanced technologies across three field deployments.

Session No.	Equipment	Camera Specs	Environmental Conditions	Challenges	Tech. Integration
**1st Deployment**	GoPro 5, GoPro 8, Samsung A7, iPad 6	720p @ 240 fps, narrow FOV, auto shutter/ISO, flat colour profile	Overcast with light rain	Mounting instability, device overheating	Baseline setup: groundwork for multi-angle capture
**2nd Deployment**	GoPro 5 and 8 (mounted front and rear on BTM)	Same specs as above	Sunny, clear visibility	Battery drainage, thermal management	Consistent multi-perspective pin tracking
**3rd Deployment**	GoPro 9, iPhone 13 Pro with LiDAR + GPS	GoPro 9: 1080p @ 240 fps; iPhone: LiDAR and 3D capture	Dark, rainy morning; low light	Waterproofing, power sustainment, and GPS stabilisation	Integrated 3D spatial capture, GPS tagging, 5G for remote sync

**Table 4:** Comparison of classification performance and real-time deployment metrics across GPU, CPU, and simulated Jetson Nano configurations. Jetson Nano results are based on FP16 TensorRT inference emulated on a downclocked RTX 4060 to reflect embedded deployment conditions.

Metric	GPU (RTX 4060, Laptop)	CPU (Intel i7-11800H)	Jetson Nano (Simulated)	Notes
Accuracy (%)	92.3	92.3	~91.1	Five-fold cross-validation (macro avg.)
F1 Score (Macro Avg.)	91.0	91.0	~89.6	Minor performance drop due to low-power constraints
Precision/Recall	High on Safe/Partial	Lower on Ambiguous	Similar pattern	Temporal smoothing improved recall for Full and Ambiguous classes
Inference Latency (ms/frame)	9.5	26.1	~48.3	FP16 mode; simulated via TensorRT and downclocked RTX 4060
Model Size (MB)	2.9	2.9	2.9	Suitable for embedded platforms
Memory Usage (MB)	~180	~210	~290	Based on peak usage during batch inference (batch size = 32)
Power Consumption	~45W	~65W	~5–10W	Jetson TDP inferred from NVIDIA documentation (low-power mode)

Note: Jetson Nano figures were obtained through controlled FP16 inference using TensorRT on a desktop GPU with matched clock/power limits. Results align with published benchmarks for MobileNet and SNN-class models.

**Table 5:** Cross-validation performance (accuracy, precision, recall, and F1 score) for all four alignment classes.

Class	Accuracy (%)	Precision (%)	Recall (%)	F1 Score (%)
Safe	94.1	93.5	94.8	94.1
Partial	90.2	88.7	89.9	89.3
Full Misalignment	92.6	91.1	90.5	90.8
Ambiguous	87.4	84.6	86.1	85.3
Macro Average	91.1	89.5	90.3	89.9

**Table 6:** Deployment benchmarks showing inference time, runtime memory usage, and model size across RTX 4060 GPU and Intel i7 CPU platforms (measured) and Jetson Nano (simulated). Power usage values are estimated based on platform specifications.

Metric	RTX 4060 GPU	Intel i7 CPU	Jetson Nano (Simulated)
Average Inference Time	9.5 ms/frame	26.1 ms/frame	48.3 ms/frame
Memory Usage (Runtime)	~180 MB	~210 MB	~290 MB
Model Size (on disk)	2.9 MB	2.9 MB	2.9 MB
Estimated Power Usage	~45 W	~65 W	~5–10 W

**Table 7:** Temporal fusion comparison: F1 scores across pin alignment classes for different aggregation strategies. Sequence-aware models, particularly the GRU-based readout, outperform frame-wise classification by improving consistency on ambiguous and partially misaligned cases, yielding a macro F1 gain of up to 2.6%.

Method	“Safe” (%)	“Partial” (%)	“Out” (%)	“Ambiguous” (%)	Macro F1 (%)
Frame-wise Only	94.1	89.3	90.8	85.3	89.9
Majority Voting	95.2	90.5	91.4	87.2	91.1
EMA Smoothing	95.6	91.2	91.8	88.1	91.7
GRU Readout (Test)	96.1	91.7	92.4	89.6	92.5

**Table 8:** Comparison of different spike encoding strategies evaluated on classification F1 score, average inference latency, and spike density. Latency coding (TTFS) achieved the best balance of accuracy and computational efficiency, while rate coding incurred higher spike activity. The analogue input bypassed spiking altogether but underperformed compared to both spike-based variants. N/A means Not Applicable.

Encoding Scheme	F1 Score (%)	Latency (ms)	Spike Density (%)
Latency (TTFS)	91.1	9.5	8.1
Rate Coding	89.3	11.8	21.6
Analogue Input (No Spike)	88.7	10.2	N/A

**Table 9:** Comparative evaluation of the proposed SIFT-SNN framework against traditional and modern CNN- and transformer-based models across macro F1 score, model size, and inference latency. Latency values are measured on the same evaluation platform for all models. Edge deployment suitability and interpretability are assessed qualitatively based on model size, computational demand, and architectural characteristics.

Model	Macro F1 Score (%)	Model Size (MB)	Inference Latency (ms/Frame)	Power Suitability (Edge)	Interpretability
SIFT-SNN	91.1	2.9	9.5	Yes	High
ResNet-50	91.4	98	34.0	No	Low
MobileNetV2	89.7	14	22.0	Limited	Low
EfficientNet-B0	90.2	20	28.0	Limited	Low
ConvNeXt-T	91.6	29	36.5	No	Low
ViT-B/16	92.0	85	41.0	No	Very Low

## Data Availability

The data presented in this study are available on request from the corresponding author due to security or privacy constraints related to access to infrastructure.

## Abbreviations

The following abbreviations are used in this manuscript:

AUT	Auckland University of Technology
BTM	Barrier Transfer Machine
CNN	Convolutional Neural Network
CLAHE	Contrast Limited Adaptive Histogram Equalisation
EMA	Exponential Moving Average
F1	F1 Score (harmonic mean of precision and recall)
FPS	Frames Per Second
GRU	Gated Recurrent Unit
IoT	Internet of Things
LIF	Leaky Integrate-and-Fire
MCB	Movable Concrete Barrier
MLP	Multi-Layer Perceptron
NZTA	New Zealand Transport Agency
ROI	Region of Interest
SIFT	Scale-Invariant Feature Transform
SNN	Spiking Neural Network
TTFS	Time-To-First-Spike
