# Correction: Rathee et al. SIFT-SNN for Traffic-Flow Infrastructure Safety: A Real-Time Context-Aware Anomaly Detection Framework. *J. Imaging* 2026, *12*, 64

**DOI:** 10.3390/jimaging12100467

**Published:** 2026-09-24

**Authors:** Munish Rathee, Boris Bačić, Maryam Doborjeh

**Affiliations:** 1 School of Engineering, Computer and Mathematical Sciences, Auckland University of Technology, Auckland 1010, New Zealand; 2 Knowledge Engineering and Discovery Research Innovation, Auckland University of Technology, Auckland 1010, New Zealand

## Error in Table

In the original publication [1], there was a mistake in Table 6 as published. To match Table 4, statistics in Table 6 should be changed as follows:

- CPU latency: change 17.3 ms to 26.1 ms;
- Jetson Nano (simulated) latency: change 28.4 ms to 48.3 ms;
- Model size: change 8.3 MB to 2.9 MB;
- Memory usage: change 1.1 GB to ~180 MB, 1.4 GB to ~210 MB, and 840 MB to ~290 MB;
- Estimated power usage: change 25 to ~65 W and 5–7 range to ~5–10 W.

The corrected Table 6 appears below.

**Table 6:** Deployment benchmarks showing inference time, runtime memory usage, and model size across RTX 4060 GPU and Intel i7 CPU platforms (measured) and Jetson Nano (simulated). Power usage values are estimated based on platform specifications.

Metric	RTX 4060 GPU	Intel i7 CPU	Jetson Nano (Simulated)
Average Inference Time	9.5 ms/frame	26.1 ms/frame	48.3 ms/frame
Memory Usage (Runtime)	~180 MB	~210 MB	~290 MB
Model Size (on disk)	2.9 MB	2.9 MB	2.9 MB
Estimated Power Usage	~45 W	~65 W	~5–10 W

In the original publication, there was a mistake in Table 9. This revision is needed so that all textual references are consistent with the corrected deployment values already reported in Table 4 and the revised Table 6. The correct Table 9 appears below.

**Table 9:** Comparative evaluation of the proposed SIFT-SNN framework against traditional and modern CNN- and transformer-based models across macro F1 score, model size, and inference latency. Latency values are measured on the same evaluation platform for all models. Edge deployment suitability and interpretability are assessed qualitatively based on model size, computational demand, and architectural characteristics.

Model	Macro F1 Score (%)	Model Size (MB)	Inference Latency (ms/Frame)	Power Suitability (Edge)	Interpretability
SIFT-SNN	91.1	2.9	9.5	Yes	High
ResNet-50	91.4	98	34.0	No	Low
MobileNetV2	89.7	14	22.0	Limited	Low
EfficientNet-B0	90.2	20	28.0	Limited	Low
ConvNeXt-T	91.6	29	36.5	No	Low
ViT-B/16	92.0	85	41.0	No	Very Low

## Text Correction

There were several errors in the original publication. These revisions are needed so that all textual references are consistent with the corrected deployment values already reported in Table 4 and the revised Table 6.

A correction has been made to the second paragraph of Section 5.2, Comparison with Traditional and Deep Learning Methods:

By contrast, the SIFT-SNN system achieves comparable performance (91.1% macro F1) while maintaining a lightweight footprint (2.9 MB) and low inference latency (9.5 ms), making it well-suited for edge deployment.

A correction has been made to the second paragraph of Section 5.5, Deployment Readiness and Edge Viability:

The Jetson Nano simulation achieved an inference latency of approximately 48.3 ms per frame, representing the projected embedded performance under the simulated constrained-hardware configuration.

A correction has been made to the third paragraph of Section 5.5, Deployment Readiness and Edge Viability:

The pipeline’s compact model size (2.9 MB) and low runtime memory usage, ranging from approximately 180 MB to 290 MB across the evaluated configurations, further reinforce its suitability for embedded systems.

A correction has been made to the Section 5.7, Contribution to Neuromorphic and Safety AI Research, Item 3: Operational neuromorphic deployment:

Our work enables neuromorphic vision to transition from lab environments to embedded systems. The system’s 9.5 ms inference on GPU and approximately 48.3 ms latency under Jetson Nano simulation demonstrate low-latency operation across heterogeneous deployment configurations and support the feasibility of field deployment which is particularly important for near-future AI computing expectations as well as in energy-constrained scenarios, such as mobile bridge inspection platforms.

A correction has been made to the third paragraph of Section 6, Conclusions:

Its architecture, featuring latency-coded spikes and LIF dynamics, demonstrates high efficiency and interpretability, with simulated Jetson Nano performance of approximately 48.3 ms per frame and a compact model footprint of 2.9 MB.

The authors state that the scientific conclusions are unaffected. This correction was approved by the Academic Editor. The original publication has also been updated.
